# New insight into Alzheimer’s disease: small molecule-Aβ in the brain transport and peripheral organ clearance

**DOI:** 10.3389/fragi.2026.1921485

**Published:** 2026-09-29

**Authors:** Tianci Ma, Yongyi Li, Yuting Ding, Shijie Liu, Chuanhao Gao, Xiang Li, Zhiqian Tong, Yan Lv

**Affiliations:** 1 Clinical Medical College, Hainan Medical University, Haikou, Hainan, China; 2 Proactive Health and Aging Medicine of Clinical Research and Translational Center, Beijing Geriatric Hospital, Beijing, China; 3 Neurology Department, Hainan General Hospital, Affiliated Hainan Hospital of Hainan Medical University, Haikou, Hainan, China

**Keywords:** Alzheimer’s disease, brain-peripheral transport and 1 clearance, melecular mechanism, therapeutic drugs, β-Amyloid (Aβ)

## Abstract

Alzheimer’s disease (AD) represents one of the most prevalent neurodegenerative disorders without approved disease-modifying therapies. A major contributing factor is that, for decades, drug development has focused almost exclusively on repairing or removing lesions within the brain itself. Yet a growing body of work now points to AD as a systemic disorder, rooted in a breakdown of whole-body Aβ homeostasis. Nevertheless cerebral Aβ transport and peripheral Aβ clearance are commonly investigated as independent processes, and the coordinated interplay… remains insufficiently characterized. This review bridges the current research gap by systematically elaborating the full trafficking cascade of brain-derived Aβ from intracerebral drainage to peripheral elimination. Four major intracranial clearance pathways are comprehensively summarized, including the blood-brain barrier, blood-cerebrospinal fluid barrier, arachnoid granulation venous outflow, and meningeal lymphatic/glymphatic system. The peripheral clearance processes of circulating Aβ in the blood, liver, kidney, and gastrointestinal tract are further illustrated. A broad spectrum of emerging therapeutic strategies targeting Aβ transport and clearance is also reviewed, including specific pathway modulators, microglia regulators, Aβ aggregation inhibitors, natural multi-target compounds, ultrasound-mediated blood-brain barrier opening, and exosome-based intervention approaches. When we compare the relative contributions of central versus peripheral clearance, Comparative analysis of central and peripheral Aβ clearance indicates that the liver appears to dominate the peripheral elimination of Aβ, yet the real driver of cerebral Aβ deposition is not the failure of any single route, but rather the loss of synergy among multiple pathways. This mechanistic framework explains why purely brain-centric therapeutics are unlikely to correct a systemic Aβ imbalance. Instead, future translational efforts should give higher priority to combination regimens that simultaneously facilitate intracerebral drainage and bolster peripheral organ clearance. This review further discusses current hurdles and emerging opportunities in systemic AD drug discovery, with the aim of offering a unified conceptual framework-one that providing a unified conceptual framework for understanding AD pathogenesis and facilitating the exploration of novel therapeutic paradigms.

## Graphical Abstract

**Figure:**
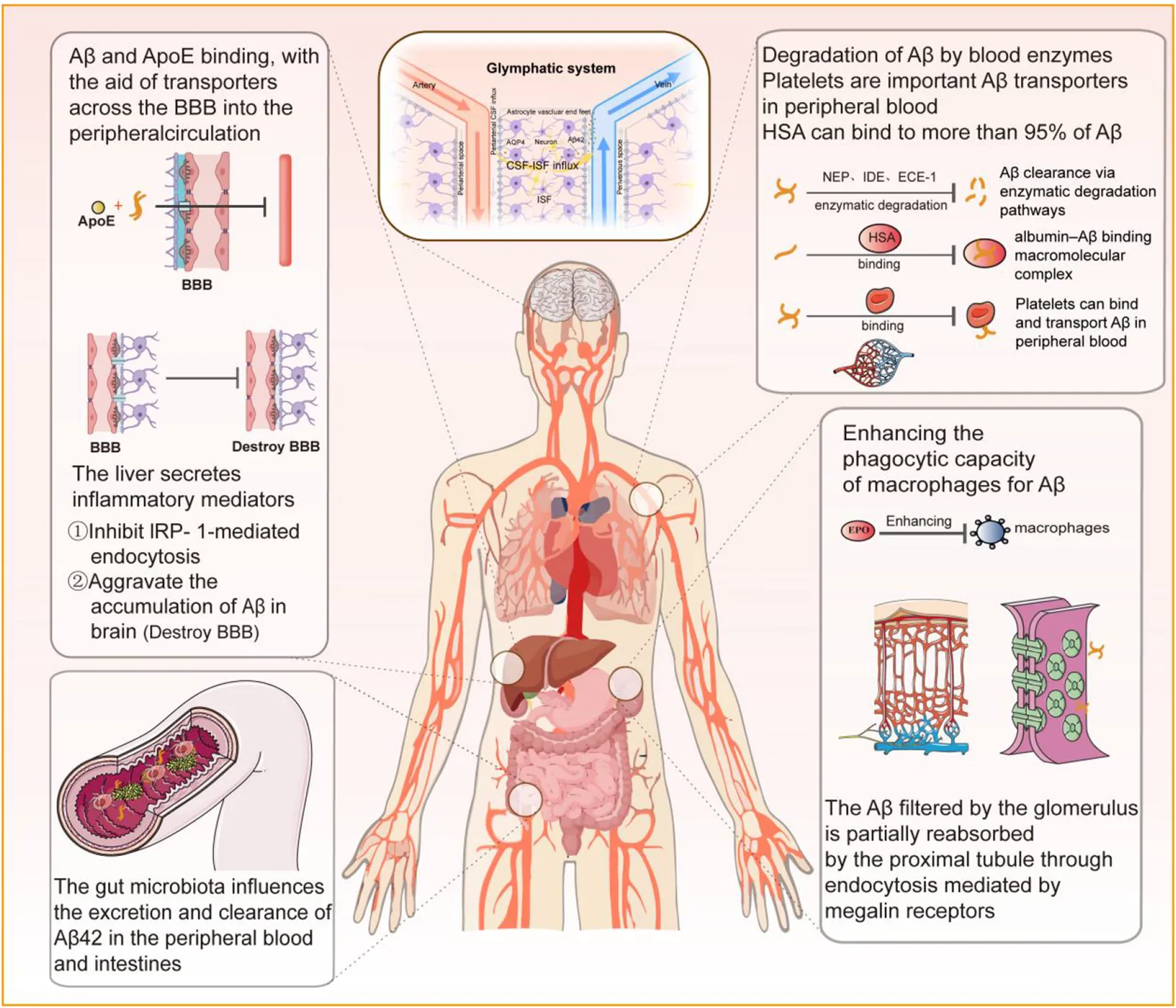

## Highlights

- The limited effectiveness of current clinical drugs remains a major challenge in managing AD.
- The idea that drugs must target the brain to treat brain disorders should be reevaluated, since AD is a systemic disease.
- Aβ degradation involves CSF-mediated brain transport and clearance by peripheral organs.
- The lack of an integrated “transport-clearance” regulatory network impedes understanding AD and developing new therapies.

## 1 Introduction

As a globally prevalent neurodegenerative disease, Alzheimer’s disease (AD) is characterized by an insidious pathological process, a narrow clinical intervention window, and limited efficacy of existing therapeutic drugs. It has become a major public health concern that seriously threatens the health and quality of life of the elderly. Currently, the abnormal accumulation of β-amyloid (Aβ) in the brain is widely recognized as the initiating core event of AD pathogenesis (Selkoe and Hardy, 2016; Busche and Hyman, 2020; Hardy and Higgins, 1992). The maintenance of its systemic homeostasis depends on the synergistic regulation between the central nervous system (CNS) and the peripheral system, and the dysfunction of the entire “intracerebral transport-peripheral clearance” cascade is a key pathological mechanism underlying Aβ accumulation (Zlokovic, 2011; Zhang H. et al., 2023; Bell et al., 2007; Yang et al., 2025).

Despite significant progress in Aβ-related research in recent years, clinical translation faces a core bottleneck: most existing studies focus in isolation on the central Aβ transport pathways (such as the blood-brain barrier and cerebrospinal fluid circulation) or the clearance function of a single peripheral organ (such as the liver and kidneys), lacking systematic integration and mechanistic elaboration of the synergistic regulatory network across the entire “transport-clearance” cascade. This fragmented research model hinders a deep understanding of “central-peripheral crosstalk” in AD, complicating the identification of key Aβ accumulation nodes. It also limits the development of new targeted therapies, as most drugs fail due to poor efficacy or safety, focusing only on single pathological aspects without addressing the need for transport pathway repair and enhanced peripheral clearance (Zlokovic, 2011; Shi et al., 2024).

Based on this, systematically sorting out the core mechanisms of Aβ brain-peripheral transport and peripheral clearance, and integrating multi-dimensional intervention strategies, are of great theoretical and clinical significance for breaking through the predicament of AD treatment (Khowdiary et al., 2025). This review focuses on the “brain-peripheral crosstalk network” that regulates Aβ homeostasis. It details the molecular mechanisms and dysfunctions in four key pathways for Aβ transport from the brain to the periphery: the blood-brain barrier, cerebrospinal fluid-blood barrier, arachnoid granule, and lymphatic pathways. It highlights the roles of transport molecules like LRP1, RAGE, and AQP4. Additionally, it discusses how the peripheral system (blood, liver, kidneys, gastrointestinal tract) works together to clear Aβ, emphasizing the importance of multi-organ interaction in maintaining Aβ balance. It reviews therapeutic strategies like transport repair, peripheral clearance enhancement, and microglia regulation, focusing on small-molecule drugs, biological agents, ultrasound interventions, and exosome carriers, while analyzing their mechanisms, research progress, and key challenges (Figure 1).

**FIGURE 1:**
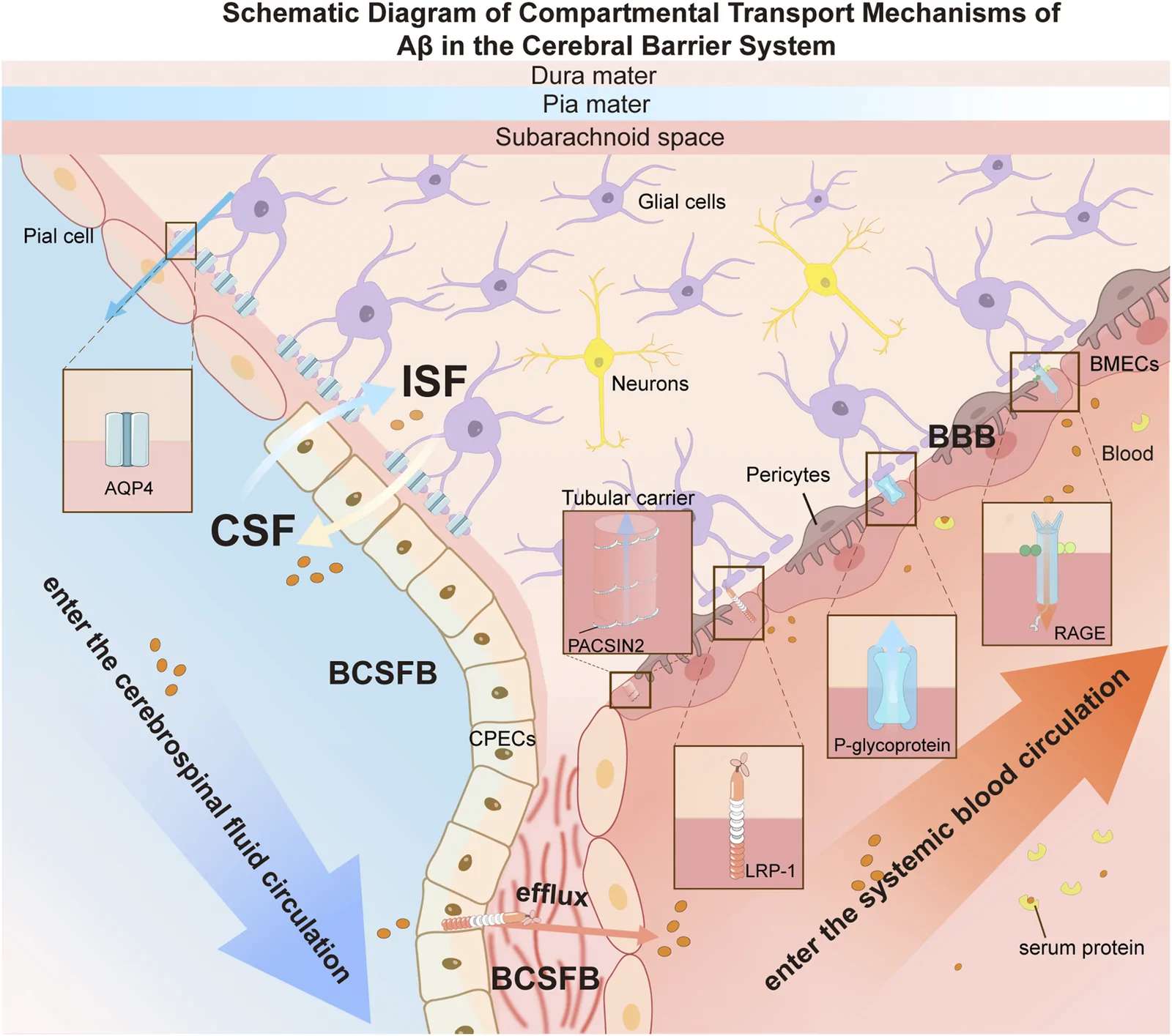
This schematic diagram is constructed based on three core compartments: brain parenchyma, vascular blood side, and cerebrospinal fluid (CSF) side. It illustrates the spatial distribution of four key transporters-LRP1, RAGE, AQP4, and P-glycoprotein-and delineates their respective directional transport activities toward β-amyloid (Aβ).

## 2 Aβ transport in the brain of AD

### 2.1 Blood-brain barrier (BBB) pathway

As a dynamic platform separating the brain parenchyma from the peripheral blood circulation, the blood-brain barrier (BBB) plays a central role in regulating central Aβ levels (Kadry et al., 2020; Abbott et al., 2010). This sophisticated system not only restricts the passive diffusion of Aβ through a physical barrier, but also actively regulates the bidirectional transport of Aβ through a variety of specific transporters, maintaining its dynamic balance in the brain (Donahue et al., 2006; Deane et al., 2009; Deane et al., 2004; David, 2004).

#### 2.1.1 Transcellular transport system

The clearance of Aβ by the BBB mainly relies on receptor-mediated transcellular transport. In 2000, a pioneering study published in The Journal of Clinical Investigation found that low-density lipoprotein receptor-related protein 1 (LRP1) is located on both the luminal (blood-facing) and abluminal (brain-facing) membranes but primarily on the abluminal side, responsible for binding Aβ and promoting its efflux from the brain parenchyma to the peripheral blood (Shibata et al., 2000). LRP1-mediated transport follows a specific molecular pathway: after binding to Aβ, LRP1 clusters and shuttles through a PACSIN2 (also known as syndapin-2)-dependent non-classical transcellular pathway, forming tubular carriers that avoid lysosomal degradation and achieve efficient Aβ clearance (Leite et al., 2022; Tian et al., 2020). The efficiency of this transport mechanism is precisely regulated by the expression level and distribution of LRP1. In the early stages of AD, the colocalization of LRP1 and Aβ is significant, actively participating in clearance (Shibata et al., 2000; Ramanathan et al., 2015); however, with disease progression, the expression of LRP1 on both the luminal (blood-facing) and abluminal (brain-facing) membranes but primarily on the abluminal side is significantly downregulated (Donahue et al., 2006; Ramanathan et al., 2015; Silverberg et al., 2010; Hsu et al., 2021; Hsu et al., 2019), leading to decreased clearance capacity and increased perivascular Aβ deposition.

Meanwhile, the ATP-binding cassette (ABC) transporter superfamily, particularly P-glycoprotein (P-gp), is also involved in the BBB efflux of Aβ (Cirrito et al., 2005; Kuhnke et al., 2007; McCormick et al., 2021). A 2012 study first confirmed impaired P-gp function in the living human brain of AD patients (van Assema et al., 2012), and its functional inhibition leads to increased Aβ accumulation in the brain, confirming its role in Aβ clearance (David, 2004). A 2018 study found that in addition to P-gp, other members of the ABC transporter family (such as BCRP and MRPs) are also involved in Aβ clearance, collectively forming an “ABC transporter network” for Aβ efflux (Storck et al., 2018). These transport systems cooperate with each other to form the main molecular basis for Aβ clearance by the BBB.

#### 2.1.2 Balance between influx and efflux

Corresponding to the efflux mechanism, as early as 2003, a study confirmed that the receptor fo advanced glycation end products (RAGE) mediates the influx of peripheral Aβ into the brain (Deane et al., 2003), a process that is particularly critical in the pathological development of AD. The expression of RAGE in BBB endothelial cells is upregulated in the early stages of AD, leading to more peripheral Aβ entering the brain parenchyma and exacerbating deposition (Miller et al., 2008). Under normal conditions, the LRP1-mediated efflux and RAGE-mediated influx maintain a dynamic balance (Donahue et al., 2006; Deane et al., 2004); however, during the progression of AD, this balance is disrupted, with decreased LRP1 expression and increased RAGE expression, resulting in reduced net efflux and Aβ accumulation in the brain (Donahue et al., 2006; Deane et al., 2004) (Figure 2; Table 1).

**FIGURE 2:**
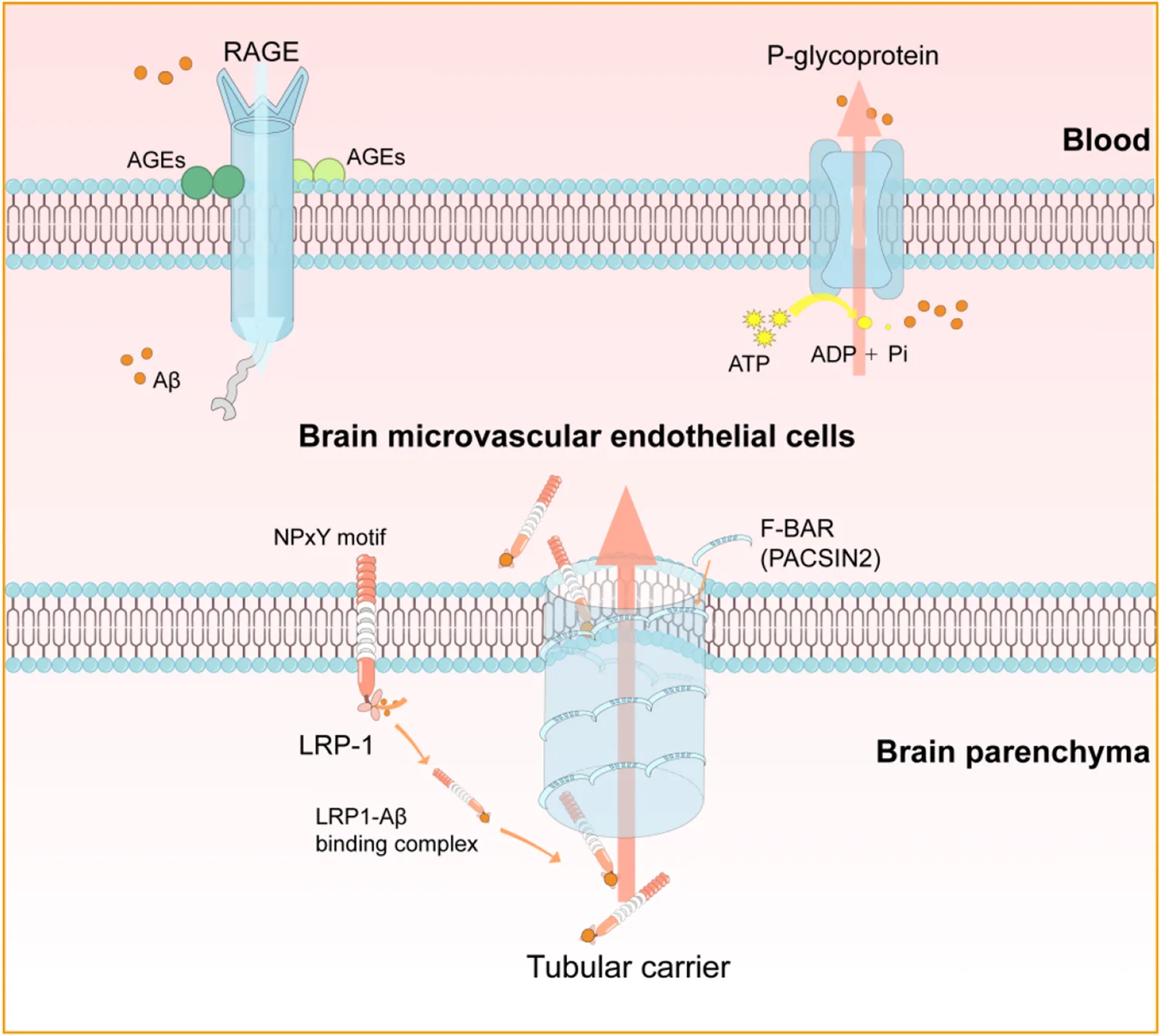
Aβ binds to the brain microvascular endothelial cells.

**TABLE 1:** Expression, function and Alzheimer’s disease-associated alterations of key Aβ transporters at the blood-brain barrier.

Transporters	Expression location	Function	Changes in AD	Ref.
LRP1	Abluminal (brain-facing) surface of brain microvascular endothelial cells	Mediates the efflux of Aβ from the brain parenchyma to the blood	The expression level of LRP1 is significantly downregulated	Storck et al. (2018)
RAGE	Luminal (blood-facing) surface of brain microvascular endothelial cells	Mediates the influx of blood Aβ into the brain parenchyma	Expression is upregulated	Deane et al. (2003)
P-gp	Luminal (blood-facing) surface of brain microvascular endothelial cells	Involved in Aβ efflux, ATP- dependent	Expression level is significantly reduced and part of it is degraded	Storck et al. (2018)
PACSIN2	Brain microvascular endothelial cells	Promotes the non-classical transcellular pathway of LRP1 and avoids lysosomal degradation.	Expression may be abnormal	D et al. (2022); Tian et al. (2020), Chen et al. (2025)

#### 2.1.3 Research perspectives

Since 2006, it has been confirmed that changes in the distribution and expression of Aβ transporters in the BBB of AD patients are key factors leading to impaired clearance function (Donahue et al., 2006). A study by Professor Yongmei Li’s team at West China Hospital found that in 12-month-old AD model mice, Aβ deposition on the BBB’s vascular side increased, while LRP1 and Aβ colocalization decreased, indicating reduced LRP1-mediated clearance as the disease progresses. Additionally, LRP1 and PACSIN2 levels in blood vessels decreased, and Rab5 levels increased, suggesting endosomal dysfunction and a reduced functional LRP1 pool for Aβ clearance.

Emerging nanomedicine strategies, like the LRP1-targeted polymer vesicles (A40-POs), show promise in addressing pathological changes (Chen et al., 2025; Deng et al., 2025). These vesicles enhance the PACSIN2-dependent transport pathway, precisely regulate ligand-receptor interactions, increase LRP1 expression, and significantly reduce brain Aβ levels by nearly 45% within 2 h, while boosting plasma Aβ levels by 8-fold. This approach not only improves Aβ clearance but also restores cognitive function for up to 6 months by repairing BBB transport, offering a novel approach for AD treatment.

### 2.2 Blood-cerebrospinal fluid barrier (BCSFB) pathway

In 2005, a study first demonstrated that the cerebrospinal fluid-blood barrier constitutes another important pathway for Aβ clearance, mainly achieving bidirectional transport of Aβ through the choroid plexus, a special structure (Crossgrove et al., 2005). While the fenestrated choroid plexus allows some fluids to go through, thigh junctions located on the apical side of these epithelial cells prevent permeation of most macromolecules. This unique anatomical feature makes it a key platform for material exchange between CSF and blood (Balusu et al., 2016; Hladky and Barrand, 2016).

#### 2.2.1 Bidirectional Aβ transport in the choroid plexus

As the main site of CSF production, the choroid plexus produces approximately 80% of CSF, with the remaining 20% coming from plasma ultrafiltrate across the BBB (Hladky and Barrand, 2016). The choroid plexus not only produces CSF but also actively participates in the Aβ clearance process (Crossgrove et al., 2005; Hladky and Barrand, 2016). Studies have shown that LRP1, LRP2, and P-gp are expressed on the basolateral side (blood-facing side) of choroid plexus epithelial cells and P-gp is primarily localized on the apical (cerebrospinal fluid-facing) membrane of brain choroid plexus epithelial cells, which can mediate the transport of Aβ from CSF to blood (Balusu et al., 2016; Asante and Barger, 2025; Pascale et al., 2011). In addition, as a biosensor, the choroid plexus can respond to changes in Aβ levels in CSF, regulate its transport activity, and maintain the stability of the CSF environment (Crossgrove et al., 2005; Pascale et al., 2011; Alvira-Botero and Carro, 2010; Duarte et al., 2020).

Notably, the transport of Aβ by the choroid plexus is bidirectional: it can not only clear Aβ from CSF to blood but also allow Aβ from blood to enter CSF (Crossgrove et al., 2005; Pascale et al., 2011; Alvira-Botero and Carro, 2010). This bidirectional transport characteristic (with efflux approximately 33% higher than influx) makes the choroid plexus play a complex role in maintaining Aβ balance in CSF. Clinical observations have found that Aβ42 levels in the CSF of AD patients are decreased, which may reflect increased Aβ deposition in the brain and/or impairment of clearance pathways, among which choroid plexus dysfunction may be an important factor (Crossgrove et al., 2005; Vlachos et al., 2012; Solár et al., 2020; González-Marrero et al., 2015) (Figure 3).

**FIGURE 3:**
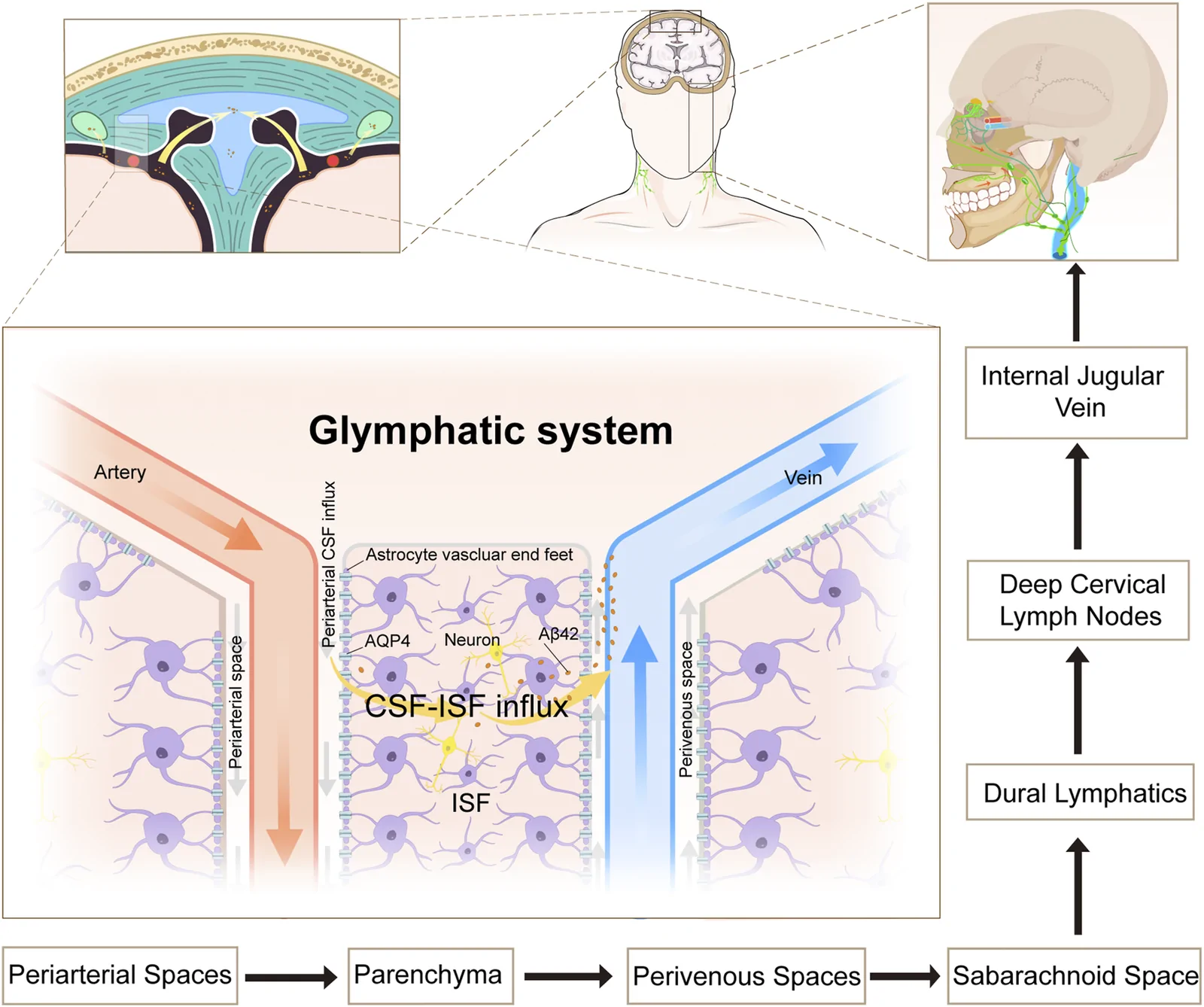
Schematic diagram of the Aβ excretion pathway via the cerebral lymphatic system. Guided by cerebrospinal fluid (CSF) drainage, Aβ is excreted from the perineuronal space into the subarachnoid space, then passes through the dural lymphatic vessels and exits the cranial cavity via the foramina at the skull base. The cerebral lymphatic system is a crucial pathway for Aβ clearance; its obstruction, damage, or absence can lead to Aβ deposition, thereby exacerbating the progression of Alzheimer’s disease (AD).

#### 2.2.2 Dynamic circulation of cerebrospinal fluid

The flow of CSF provides a natural clearance pathway for Aβ. After being produced by the choroid plexus, CSF flows sequentially through the lateral ventricles, third ventricle, and fourth ventricle, and then enters the subarachnoid space (SAS) (Donahue et al., 2006; Iliff et al., 2012; May et al., 1990; Fishman, 2002). During this circulation process, CSF exchanges substances with interstitial fluid (ISF), carries metabolic wastes including Aβ, and finally returns to the peripheral circulation through multiple pathways (Huang et al., 2024; Xie et al., 2013). CSF flow depends not only on the pressure difference between production and absorption but also on various kinetic factors such as arterial pulsation and respiratory rhythm (Yildiz et al., 2017; Iliff et al., 2013; Vinje et al., 2019).

The dynamic circulation characteristic of CSF makes it an ideal waste clearance system. Studies have found that CSF flow rate is closely related to Aβ clearance rate, and decreased flow rate may lead to Aβ accumulation in the brain (Iliff et al., 2012; Li Y. et al., 2022; Shang et al., 2024). With aging and AD progression, CSF production decreases and circulation slows down, which may partially explain the decreased Aβ clearance efficiency in AD patients (Fishman, 2002; Silverberg et al., 2003).

#### 2.2.3 Clinical research evidence

Evidence supporting the role of the BCSFB in Aβ clearance comes from multiple clinical studies. A study using stable isotope labeling kinetics (SILK) technology showed that abnormal exchange of Aβ between CSF and blood is closely related to AD pathology (Elbert et al., 2022). Researchers tracked the kinetics of different Aβ isoforms (Aβ38, Aβ40, Aβ42) in CSF and blood through bolus injection labeling, and found that these kinetic parameters changed in AD patients, reflecting dysfunction of clearance pathways.

Another study focused on the correlation between CSF and blood Aβ levels and found that changes in blood Aβ kinetics can reflect abnormal Aβ metabolism in the central nervous system, which provides the possibility for the development of AD blood biomarkers (Ovod et al., 2017). These studies collectively indicate that the BCSFB is not only a physical channel for Aβ clearance but also an important window reflecting the pathological process of AD.

### 2.3 Arachnoid granulation-mediated CSF venous drainage pathway

#### 2.3.1 Anatomical structure and functional characteristics

Arachnoid granulations (also known as arachnoid villi or Pacchioni’s corpuscles), finger-like protrusions projecting into the dural venous sinuses, are important anatomical channels for the direct drainage of cerebrospinal fluid (CSF) into the peripheral blood and play a crucial role in the transport of Aβ from the CSF to the periphery. Unlike the active transport mechanisms of the BBB and CSF-BBB, this system is mainly driven by physical pressure differences (Chen et al., 2015).

Arachnoid granulations are widely distributed around the dural venous sinuses, such as the superior sagittal sinus, transverse sinus, and sigmoid sinus. They penetrate the dura mater and protrude into the lumen of the venous sinuses (Glimcher et al., 2008). Arachnoid granulations have an outer cystic layer and an inner core made of loose connective tissue and arachnoid cells, with a surface of flattened arachnoid cells connected to the venous sinus endothelium. The venous sinus has flowing blood, while the subarachnoid space holds static CSF, creating a pressure difference that drives Aβ-carrying CSF drainage into the blood (Proulx, 2021).

CSF drains through arachnoid granulations primarily due to the hydrostatic pressure difference between CSF and venous blood, not active transport. When CSF pressure exceeds venous sinus pressure, it flows into the venous system via the one-way valve-like structure of arachnoid granulations, without energy use (Hasegawa et al., 1997). Arachnoid granulations selectively clear solutes, removing 40%–80% of macromolecular proteins, including Aβ peptides. They may clear different Aβ isoforms, like Aβ40 and Aβ42, with varying efficiency. Aβ42, which aggregates more easily, may be cleared less effectively, contributing to its accumulation in Alzheimer’s disease (Deane et al., 2009; Xie et al., 2013).

#### 2.3.2 Aβ clearance mechanism and pathological significance

In summary, dysfunction of arachnoid granulations is closely related to AD pathology. Although there are some individual differences, overall, with aging, arachnoid granulations undergo fibrosis or calcification, leading to decreased CSF drainage function (Suzuki et al., 2021). Especially in the elderly AD patient population, patients may be accompanied by morphological changes of arachnoid granulations, which can seriously affect the CSF drainage process in severe cases. This deterioration of structure and function will exacerbate Aβ accumulation in the brain, further impair CSF drainage function, and thus form a vicious circle (Roche and Warner, 1996). Currently, AD is generally regarded as “a manifestation of systemic diseases in the brain”, indicating that its progression is destined to be observed from multiple dimensions and intervened with multiple methods. Promoting research on this pathological process will become an inevitable trend in future AD pathological research.

### 2.4 Lymphatic-associated pathway

Despite advances in understanding the mechanisms of brain-derived Aβ production and clearance, mainstream drugs like Aducanumab, Lecanemab, and Donanemab, which target the central microglial system, remain ineffective. Consequently, the medical community is increasingly focusing on lymphatic-associated pathways for Aβ clearance. The discovery of the Glymphatic System by Professor Maiken Nedergaard’s team in 2012 and the confirmation of dural lymphatic vessels in 2015 by Louveau and Aspelund’s teams have revolutionized neuroscience (Iliff et al., 2012; Louveau et al., 2015). These findings, using advanced imaging technology, have transformed the understanding of brain immune surveillance and waste clearance. The clarification of this complete “intracerebral clearance-extracerebral drainage” pathway not only reveals the potential limitations of the traditional single strategy targeting microglia but also opens up a highly promising new intervention direction for AD treatment (Yamada and Iwatsubo, 2024; Wang et al., 2024; Li G. et al., 2022), as shown in Figure 3.

#### 2.4.1 Glymphatic System

The glymphatic system is a brain waste clearance mechanism consisting of three components: the perivascular CSF inflow pathway, the CSF-ISF exchange pathway reliant on aquaporin 4 (AQP4) in astrocyte endfeet, and the perivascular ISF clearance pathway. CSF enters the brain along perivascular spaces, mixes with ISF to remove metabolic wastes like Aβ, and exits via perivenous spaces. Solutes such as β-amyloid are expelled through arachnoid granulations, meningeal lymphatic vessels, or nerves. AQP4, located in astrocyte endfeet, facilitates fluid flow and waste clearance by managing water exchange between CSF and ISF (Mestre et al., 2018).

This polar distribution depends on the dystrophin-associated protein complex (DAPC) composed of dystrophin, α/β-dystroglycan, and α-syntrophin, which collectively anchor AQP4 to perivascular endfeet (Nagelhus and Ottersen, 2013). When solute clearance function is impaired, perivascular solutes may accumulate in the basement membrane of smooth muscle cells, triggering diseases such as cerebral amyloid angiopathy (Chachaj et al., 2023; Louveau et al., 2017). In 2025, Zhou X et al. focused on the effect of long-term high-fat diet (HFD) on Aβ clearance by the glymphatic system. TGN-073, which was confirmed as an AQP4 enhancer in 2018, played a key intervention role in this process, mainly by restoring the polarity of AQP4, promoting its ability to drive fluid flow and solute clearance, alleviating lymphatic clearance disorders, and participating in the transport of brain-derived Aβ (Peng et al., 2025). Further exploration of the core conclusions regarding TGN-073 will fill the gap in the understanding of Aβ clearance mechanisms and provide key new evidence for the prevention and treatment of Aβ deposition-related disease.

#### 2.4.2 Extracerebral drainage pathway via lymphatic vessels

Dural lymphatic vessels (mLVs) are primarily found along the dural venous sinuses, such as the superior sagittal, transverse, and sigmoid sinuses. They are categorized into dorsal and basal parts based on their location. Dorsal mLVs, located along the superior sagittal and transverse sinuses, resemble initial lymphatic vessels with sealed junctions but lack valves and smooth muscle cells. Basal mLVs, found near the petrosal and sigmoid sinuses at the skull base, have larger diameters, numerous capillary branches, button-like junctions, and lymphatic valves, acting as main channels for CSF drainage (Çavdar et al., 2023; Vera Quesada et al., 2023).

Aspelund et al. (2015) found that dural lymphatic vessels absorb CSF and ISF, transporting them to deep cervical lymph nodes via skull base foramina. This was confirmed by the absence of tracer accumulation in cervical lymph nodes of transgenic mice lacking these vessels, compared to the presence of tracer in wild-type mice (Aspelund et al., 2015). Louveau et al. (2015) discovered that meningeal lymphocytes facilitate the movement of fluid and immune cells from the CSF to the deep cervical lymph nodes. They observed that many neurological diseases involve inflammation, and dural lymphatic vessels, situated primarily around the skull base and venous sinuses, offer a direct route for intracerebral waste to access the peripheral immune system (Louveau et al., 2015).

Multiple studies have confirmed that Aβ is transferred from the brain parenchyma to the cervical lymph nodes via the glymphatic system and dural lymphatic vessels. Initially, Aβ diffuses into the brain’s extracellular space, enters the glymphatic system’s perivenous spaces, merges into the subarachnoid space, and is drained through dural lymphatic vessels at the skull base to the cervical lymph nodes, eventually reaching systemic circulation. This process relies on the glymphatic system’s perivascular spaces for initial drainage and dural lymphatic vessels for final output, working together for Aβ clearance (Çavdar et al., 2023).

In addition, the cribriform plate is also an important channel for dural lymphatic vessels to drain out of the skull (Proulx, 2021). The cribriform plate is located in the horizontal part of the ethmoid bone and contains a large number of foramina, allowing olfactory filaments to extend from the olfactory bulb to the nasal mucosal epithelium. CSF can exit through the cribriform plate through three anatomical pathways (Jacob et al., 2022): 1). Perineural pathway: along the perineural spaces of the olfactory nerve sheath, reaching the lymphatic vessels of the submucosa through cellular tissue; 2). Direct pathway: along the perineural spaces of the olfactory nerve sheath, directly reaching the lymphatic vessels of the submucosa; 3). Direct lymphatic pathway (main): the subarachnoid space can directly exit the skull through the lymphatic vessels of the cribriform plate (Jacob et al., 2022; Spera et al., 2023).

The nasal lymphatic plexus is also connected to the anterior hard palate lymphatic trunk. The involvement of the hard palate lymphatic plexus in CSF outflow is attractive because continuous movements during chewing, sucking, and speaking promote CSF drainage through the lymphatic plexus to scLV-2, which converges to the submandibular lymph nodes (Jin et al., 2025). There is a lymphatic plexus around the ophthalmic vein, which is also an important channel for CSF drainage. There is a highly dense lymphatic network in the cavernous sinus region of the skull base. Meningeal lymph flows through scLV-1, exits the skull through the orbital fissure, and finally merges with the periorbital lymphatic vessels, eventually reaching the submandibular lymph nodes (Jin et al., 2025).

#### 2.4.3 Functional regulation and pathological significance

The function of the glymphatic system is regulated by various factors. The sleep-wake cycle has a particularly significant impact on it: during non-rapid eye movement (NREM) sleep, the activity of the glymphatic system is significantly enhanced, the volume of the extracellular space increases by up to 60%, and fluid resistance decreases, thereby greatly promoting the clearance of wastes such as Aβ (Hablitz et al., 2020). As people age, sleep quality worsens, with more frequent insomnia, shorter sleep duration, and increased sleep fragmentation. Elderly individuals, particularly those over 60, often experience light sleep and rarely enter deep sleep, which is crucial for effective lymphatic transport. This reduced deep sleep time may impair the brain’s ability to clear waste, raising the risk of neurodegenerative diseases and accelerating their progression. Disruptions in sleep structure and depth can significantly decrease cerebrospinal fluid clearance and protein waste removal, potentially leading to waste buildup and Aβ aggregate formation. This finding explains the potential association between sleep and AD risk: insufficient sleep may lead to Aβ accumulation by impairing glymphatic system function (Nedergaard and Goldman, 2020; Licastro et al., 2024) (Table 2).

**TABLE 2:** Intracerebral drainage routes and extracerebral efflux pathways mediating Aβ clearance.

Main characteristics	Role in Aβ clearance	Synergistic relationship with other elements	Ref.
CSF enters the brain parenchyma along perivascular spaces	Transports fresh CSF to the deep brain, providing a basis for ISF exchange	Provides a fluid source for subsequent CSF-ISF exchange and Aβ clearance	Iliff et al. (2012)
AQP4 is highly concentrated in perivascular astrocyte endfeet	Drives CSF-ISF exchange, promoting Aβ transport from the brain parenchyma to perivascular spaces	Connects perivascular inflow and perivenous outflow, serving as the core driving element of the glymphatic system.	Iliff et al. (2012)
Wastes flow out of the brain parenchyma along perivenous spaces and merge into the Subarachnoid space	Transports metabolic wastes such as Aβ from the brain parenchyma to the subarachnoid space	Undertakes Aβ drainage in the brain parenchyma, providing a pre-channel for dural lymphatic vessel transport	Iliff et al. (2012), Louveau et al. (2015)
Dural lymphatic vessel-like structures connecting the Subarachnoid space to the Extracranial lymphatic system	Drains Aβ from the subarachnoid space to the cervical lymph nodes, completing the extracerebral output of intracerebral Aβ	As a terminal channel, it forms a complete extracerebral Aβ drainage chain together with extracranial nerves and facial lymphatic system.	Aspelund et al. (2015), Louveau et al. (2015)

## 3 Aβ clearance by the peripheral system in AD

Since Glenner and Wong first identified and purified Aβ peptides in 1984, numerous studies have demonstrated that Aβ can be cleared by peripheral organs (Glenner and Wong, 1984), as shown in Figure 4.

**FIGURE 4:**
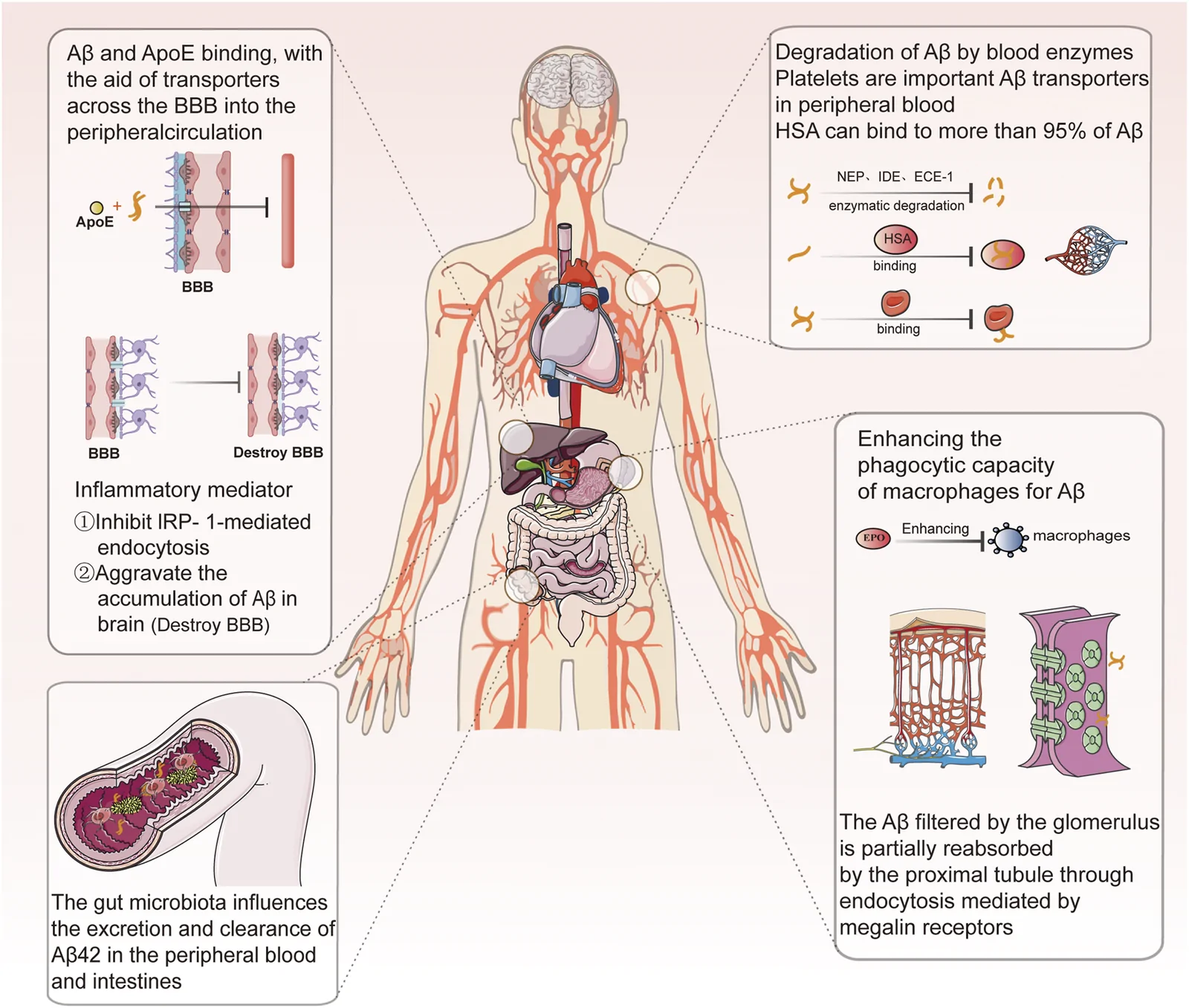
Schematic diagram of Aβ peripheral clearance and transport pathways. Current research rarely systematically investigates the synergistic Aβ clearance effects mediated by multiple peripheral organs. This schematic summarizes the core mechanisms underlying Aβ elimination in the liver, gastrointestinal tract, kidneys and circulating blood, with the aim of elucidating the coordinated organ crosstalk that combats systemic Alzheimer’s disease (AD).

### 3.1 Aβ transport and clearance in the blood

Circulating Aβ mainly exists in protein-bound forms, and its metabolic half-life differs greatly between free and bound states, which is a key easy-to-confuse point in previous studies. The half-life of free unbound Aβ in peripheral circulation is only 2.5–15 min (Hone et al., 2003; Ghiso et al., 2004), while the free Aβ concentration in the blood of Alzheimer’s disease (AD) patients is 3–4 times higher than that of healthy individuals (Sagare et al., 2007). By contrast, protein-complexed Aβ can stably exist in peripheral blood for up to 30 days, which largely relies on multiple circulating binding proteins to mediate transport and clearance.

Low-density lipoprotein receptor-related protein 1 (LRP1) on the liver cell membrane is the core receptor mediating hepatic uptake of blood-derived Aβ and acts as the primary peripheral clearance receptor for Aβ (Mohamed and Kaddoumi, 2013). Soluble LRP1 (sLRP1) has consistent biological functions with membrane-bound LRP1 and serves as a vital soluble Aβ carrier in plasma. Quantitative binding data are originally reported by Sagare et al., showing that sLRP1 binds approximately 70% of plasma Aβ_1-40_ and 90% of plasma Aβ_1-42_, and this binding effect acts as a rate-limiting barrier to prevent excessive Aβ infiltration into brain parenchyma (Sagare et al., 2007). Transthyretin (TTR) is another important circulating Aβ transporter. TTR can alleviate Aβ cytotoxicity and positively upregulate the expression level of LRP1. Meanwhile, TTR can bind circulating Aβ and deliver the complex to the liver for subsequent proteolytic degradation (Alemi et al., 2016).

As the most abundant plasma protein, human serum albumin (HSA) dominates the systemic transport of circulating Aβ and is indispensable for peripheral Aβ clearance. Relevant quantitative data show that nearly 90% of plasma Aβ binds to albumin, and only about 5% binds to circulating lipoproteins. When the Aβ concentration reaches 5 ng/mL, over 95% of Aβ molecules form stable binding complexes with albumin (Kuo et al., 2000). The albumin-Aβ macromolecular complex regulates the whole process of Aβ peripheral transport and elimination (Xie and Guo, 2020). Functioning as an endogenous Aβ aggregation inhibitor, HSA reduces the β-sheet structural proportion of Aβ monomers, blocks the assembly of long β-strand chains, reshapes Aβ conformational distribution to a low β-sheet state, changes the internal binding mode of peptide chains, and ultimately inhibits Aβ fibrillization. Such evidence indicates that reduced blood albumin levels will aggravate cerebral amyloid deposition and raise the incidence risk of AD (Kim et al., 2020).

Platelets perform dual regulatory functions in peripheral blood: they act as circulating carriers for Aβ and are also one of the major endogenous sources of systemic Aβ (Wu et al., 2021). Platelet-derived production and release of Aβ: Platelets fully express α-, β-, and γ-secretases required for APP cleavage, and store all APP metabolites (sAPPα, sAPPβ, Aβ) in intracellular α-granules. Platelet activation can significantly boost Aβ secretion; Kucheryavykh et al. reported that local Aβ concentration increases by 500-fold at vascular coagulation sites (Aljehani et al., 2024). This phenomenon suggests Aβ may be a stress-responsive molecule released during trauma, coagulation and inflammatory responses, and targeted inhibition of inflammatory cascades can reduce excessive platelet-derived Aβ release.

Reciprocal activation between Aβ and platelets: Circulating Aβ can activate platelets in turn, triggering multiple downstream signaling cascades including mitogen-activated protein kinase (MAPK) activation, cytoskeletal rearrangement, and abnormal reactive oxygen species (ROS) accumulation (Li and Liu, 2022). Activated platelets participate in cerebrovascular pathological damage in AD, destroy the integrity of the blood-brain barrier (BBB), and promote the influx of peripheral circulating Aβ into brain tissue. A large proportion of blood Aβ originates from platelets, forming stable platelet-Aβ complexes in peripheral circulation, which shuttle Aβ into the brain and accelerate AD pathological progression (Wu et al., 2021).

Enzymatic degradation in plasma is a core pathway for peripheral Aβ clearance, maintaining the dynamic balance of Aβ between the brain and blood. This clearance network includes two major links: trans-barrier Aβ efflux across the BBB and the blood-CSF barrier, and proteolytic degradation mediated by multiple soluble and membrane-bound proteases in peripheral blood (Xiang et al., 2015). The key Aβ-degrading enzymes involved mainly include neprilysin (NEP), insulin-degrading enzyme (IDE), endothelin-converting enzyme 1 (ECE-1) and angiotensin-converting enzyme I (ACE).

Neprilysin (NEP): The primary Aβ-degrading zinc metalloprotease. It is a type II transmembrane protein containing 750 amino acid residues, with the catalytic active domain located at the extracellular carboxyl terminus. NEP can degrade both monomeric and oligomeric Aβ; AD patients show significant downregulation of NEP mRNA, protein expression, and enzymatic activity in both central and peripheral compartments (Qiu et al., 1998). Insulin-degrading enzyme (IDE): An M16 family zinc metallopeptidase responsible for clearing APP-derived amyloid peptides in blood and brain. Reduced IDE expression is an independent high-risk factor for AD, and IDE cooperates with the APOE genotype and cerebral insulin metabolism to jointly regulate whole-body Aβ metabolic homeostasis (Liu et al., 2015). Endothelin-converting enzyme 1 (ECE-1): Homologous to NEP, it cleaves full-length Aβ at three independent sites to generate truncated non-toxic fragments Aβ_1-17_, Aβ_1-19_ and Aβ_20-40_. Angiotensin-converting enzyme I (ACE): Complementary to the above proteases, it accelerates the degradation of Aβ_1-40_ and Aβ_1-42_, thereby inhibiting spontaneous Aβ oligomerization and senile plaque formation (Hampel et al., 2021).

In addition to enzyme-dependent degradation, immune-mediated phagocytosis by peripheral monocytes serves as an auxiliary clearance pathway for circulating Aβ. Human peripheral monocytes are divided into three subsets according to CD14 and CD16 surface markers, all of which possess Aβ phagocytic capacity with distinct functional intensity (Leinenga et al., 2021). Intermediate monocytes (CD14^++^CD16^+^) exhibit the strongest Aβ clearance activity and highly express Aβ recognition receptors such as TREM2, CD68 and CD36. These monocytes capture soluble Aβ oligomers in blood and perivascular spaces, cross the impaired BBB to infiltrate brain parenchyma, and phagocytose insoluble fibrillar Aβ plaques. Internalized Aβ is ultimately degraded in lysosomes via lysosomal hydrolases and cathepsin proteases.

### 3.2 Kidneys in Aβ clearance

As the main excretory organ of the human body, the kidneys play a key role in Aβ clearance (Xiang et al., 2015), but their clearance efficiency is lower than that of the liver. Studies have confirmed that brain-derived Aβ can enter the peripheral circulation and be filtered, reabsorbed, and degraded by the kidneys, thereby maintaining Aβ homeostasis in the body. Decreased renal function leads to impaired peripheral Aβ clearance, which may further exacerbate the pathological process of AD through the “peripheral-central” interaction mechanism.

Clinical observations indicate that peripheral Aβ clearance is linked to renal function. A study found higher Aβ levels in the superior vena cava than in the inferior vena cava (Venous Gradient Sampling Assay in Humans: Human venous gradient sampling was performed. Blood samples were collected at multiple vascular sites to determine differences in Aβ concentrations), suggesting brain-derived Aβ is cleared by peripheral organs, including the kidneys (Rodent Tracer Biodistribution Study: Rodents were used as experimental subjects. Tracer-labeled peptides were administered, and blood and various tissue samples were harvested at serial time points to investigate the *in vivo* distribution of Aβ). The renal clearance rate of Aβ is about 12.5%, contributing up to 27.7% to brain Aβ clearance. After 90 min of Aβ injection, the liver sequesters 40% of Aβ, while the kidneys hold 5% (Radiolabeled Peptide Uptake Assay: Peptides were radiolabeled with radioisotopes. Radiometric signals were used to quantitatively evaluate transmembrane transport, cellular uptake and retention of Aβ). The liver absorbs about 65% of injected radiolabeled Aβ_1-40_ and Aβ_1-42_, with the kidneys absorbing less than 10%.

This indicates that in the peripheral Aβ clearance system, the kidneys are not the core clearance organ, and their contribution to Aβ clearance is far less than that of the liver. However, we cannot ignore the role of the kidneys in Aβ. Researchers have conducted experiments to find factors for plasma β-amyloid accumulation in kidney disease, proving that Aβ can enter the urine through glomerular filtration and then be excreted (Gronewold et al., 2016). Another study showed that serum Aβ levels are related to renal function reflected by estimated glomerular filtration rate (eGFR) and residual glomerular filtration rate (rGFR). The results of these studies have confirmed the potential of dialysis as a potential Aβ clearance therapy (Liu et al., 2015). Aβ filtered by the glomerulus is reabsorbed in the proximal tubules via megalin receptor-mediated endocytosis and broken down by enzymes like neprilysin. Additionally, kidney-secreted erythropoietin boosts macrophage phagocytosis of Aβ via the PPARγ pathway and increases degrading enzyme expression, aiding Aβ clearance.

### 3.3 Aβ degradation in gastrointestine

Aβ42 can be cleared through intestinal excretion, and the gut microbiota (GM) can affect the excretory clearance of Aβ42 in the peripheral blood and intestines (Wu S. et al., 2022). Studies have shown that the apical sodium-dependent bile acid transporter (ASBT), a receptor-like protein in intestinal epithelial cells, has an affinity for amyloid β-protein, indicating that Aβ may be reabsorbed from the intestinal contents into the systemic circulation through this receptor (Bhattacharjee and Roy, 2023), which also indirectly demonstrates the feasibility of Aβ excretion from the systemic circulation into the intestines.

The gut microbiota refers to a diverse and dynamic microbial community living in the digestive tract, which can affect the health and diseases of the host. The gut microbiota communicates with the brain through a complex bidirectional communication system-the gut-brain axis, which integrates peripheral intestinal function with brain centers of emotion and cognition through neuro-immune-endocrine mediators (Sun et al., 2020). The establishment of the gut-brain axis has revealed a clear association between the gut microbiota and cognition. Multiple experimental and clinical studies have shown the role of the gut microbiota in host cognition and its age-related dysregulation leading to neurodegeneration. Gut microbiota dysregulation leads to the secretion of amyloid and lipopolysaccharide (LPS), disrupting gastrointestinal permeability and the blood-brain barrier (Jiang et al., 2017), thereby regulating inflammatory signaling pathways, promoting neuroinflammation and nerve damage, and ultimately leading to neuronal death in Alzheimer’s disease. Probiotic supplements are associated with improved cognition in AD patients (Akbari et al., 2016), which further supports the role of the gut microbiota in AD development.

A recent study showed that Aβ peptides have antibacterial properties as an innate immune response to pathogenic microorganisms. Another study showed that bacterial amyloid has similarity to Aβ peptides in molecular mimicry, which leads to misfolding and aggregation of Aβ peptides, their seeding and spread through the gut-brain axis, and subsequent activation of microglia (Kapoor et al., 2024), (Kesika et al., 2021).

### 3.4 Liver in Aβ clearance

Peripheral clearance of circulating amyloid-β (Aβ) is predominantly driven by the liver. Organ-comparison experiments confirmed that the liver accounts for approximately 60% of peripheral Aβ clearance, with a clearance capacity significantly superior to that of the kidneys and intestines (Huang et al., 2022). Liver sinusoidal endothelial cells and hepatocytes synergistically capture blood-borne Aβ, in which LRP-1 on the hepatocyte surface serves as the rate-limiting molecule mediating this process. Quantitative isotope perfusion analysis demonstrated that during a single pass through the hepatic microcirculation, 13.9% of Aβ 42% and 8.9% of Aβ 40 in plasma were cleared. Upon entering the liver via the bloodstream, Aβ is recognized by LRP-1 and undergoes receptor-mediated endocytosis; the internalized Aβ is subsequently transported to lysosomes, where it is hydrolytically degraded by insulin-degrading enzyme (IDE) and neprilysin (NEP). The residual metabolites are then excreted into the intestine through the bile ducts, achieving complete elimination. This process constitutes the core metabolic pathway for the efflux of brain-derived Aβ.

The hepatic Aβ clearance pathway described above constitutes the efferent branch of the liver-brain axis, directing from the brain to the periphery; concurrently, the liver secretes multiple bioactive mediators into the circulation, forming a reciprocal signaling pathway through which the periphery modulates the central nervous system. Together, these two arms establish the complete bidirectional regulatory network of the liver-brain axis. Under physiological conditions, the PPARα pathway upregulates LRP-1 transcription in hepatocytes, securing a stable hepatic Aβ uptake efficiency. (Song et al., 2025), (Peng et al., 2024). Aging, Alzheimer’s disease (AD), and chronic liver diseases disrupt liver-brain axis homeostasis in a concerted manner: on the one hand, downregulation of hepatocyte LRP-1 expression directly impedes peripheral Aβ clearance; on the other hand, abnormally elevated soluble epoxide hydrolase (sEH) and dysregulated secretion of FGF-21 and ApoJ from the liver alter cerebral Aβ aggregation and microglial phagocytic capacity. Chronic liver inflammation releases IL-6 and TNF-α, which deliver a dual pathological insult-they inhibit LRP-1-mediated Aβ endocytosis in hepatocytes and simultaneously damage tight junction integrity of the blood-brain barrier, thereby exacerbating the reflux of peripheral Aβ into the brain parenchyma. This establishes a vicious cycle characterized by “insufficient peripheral Aβ clearance and increased central deposition,” amplifying the pathological damage of Alzheimer’s disease. The complete pathway through which the liver bidirectionally regulates cerebral Aβ metabolism-by clearing circulating Aβ and releasing a spectrum of circulating signaling molecules-constitutes the core feature that distinguishes the liver-brain metabolic interaction axis from other peripheral organs. The following sections will respectively elaborate on the specific molecular mechanisms by which liver-derived secretory proteins and hepatic pathological states interfere with Aβ clearance.

The liver regulates Aβ clearance by secreting proteins. With age, liver activity of soluble epoxide hydrolase (sEH) increases, degrading 14,15-EET, which normally inhibits Aβ aggregation and boosts microglial phagocytosis. 14,15-EET aids in ApoE release from astrocytes, which then activates microglial TREM2, enhancing Aβ plaque clearance. Elevated liver sEH activity, as seen in aging or Alzheimer’s, reduces 14,15-EET, disrupting its balance with Aβ and accelerating Aβ deposition (Wu et al., 2023). In addition, fibroblast growth factor 21 (FGF-21) exerts neuroprotective effects by regulating cerebral insulin sensitivity and inhibiting neuroinflammation (Liang et al., 2014); apolipoprotein J (ApoJ) is directly involved in the Aβ clearance process (Ko et al., 2022). These hepatokines collectively form a complex regulatory network that coordinates the effect of the liver on cerebral Aβ metabolism.

With the occurrence of pathological conditions such as aging, the liver’s ability to clear Aβ decreases. Decreased LRP-1 expression is the core reason for this phenomenon, which hinders the peripheral transport and clearance of Aβ (Cheng et al., 2023). Patients with non-alcoholic fatty liver disease, alcoholic liver injury, and cirrhosis have decreased liver LRP1 expression, accompanied by increased plasma Aβ levels (Weinstein et al., 2019; Weinstein et al., 2020). Early studies found that these AD risk factors can impair Aβ clearance through two mechanisms: one is inhibiting liver LRP1 expression, impairing monocyte phagocytic function, disrupting the balance of peripheral Aβ clearance, and exacerbating intracerebral Aβ deposition (Tsoy et al., 2024). The other mechanism is that liver inflammation (such as the release of IL-6 and TNF-α) inhibits LRP-1-mediated Aβ endocytosis, and at the same time disrupts BBB integrity, exacerbating Aβ influx into the brain (Song et al., 2025).

Based on these roles of the liver in Aβ, it brings many inspirations for AD treatment. It is possible to target LRP1 activation to enhance the liver’s ability to take up Aβ; regulate the activity of Aβ-degrading enzymes, such as neprilysin (NEP) and insulin-degrading enzyme (IDE), to promote Aβ degradation (Gross et al., 2024); and intervene in sEH activity through methods such as gene knockdown and gene therapy to regulate 14,15-EET levels, improving intracerebral Aβ metabolism at multiple links (Wu et al., 2023). These strategies all provide new directions and ideas for AD treatment (Figure 5).

**FIGURE 5:**
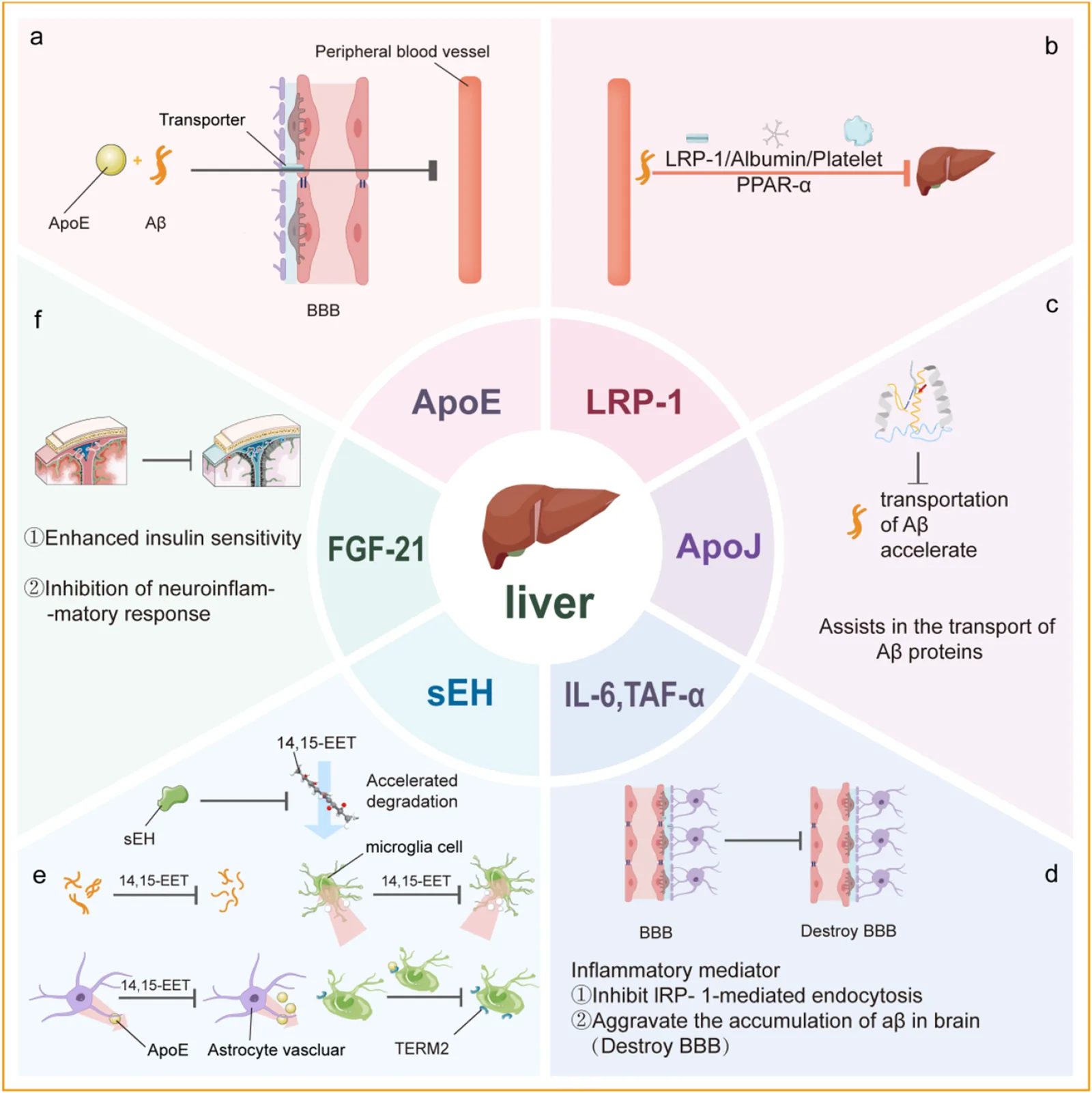
The Role of Hepatokines in Alzheimer’s Disease **(a)** ApoE mediates Aβ transport across the BBB. **(b)** LRP-1 facilitates peripheral Aβ clearance under PPAR-α modulation. **(c)** ApoJ assists the transportation of Aβ proteins. **(d)** Hepatic IL-6 and TNF-α disrupt the BBB and promote cerebral Aβ accumulation. **(e)** The sEH/14,15-EET axis modulates astrocytic and microglial activities. **(f)** FGF-21 enhances insulin sensitivity and inhibits neuroinflammation.

## 4 Therapeutic drugs for AD via Aβ brain-peripheral transport and clearance network

The research and development of Alzheimer’s disease (AD) drugs is evolving from targeting the brain alone to a broader approach that includes repairing transport pathways, enhancing peripheral clearance, and multi-target regulation. This article reviews key drugs that focus on β-amyloid (Aβ) transport between the brain and periphery, peripheral clearance, and related pathways. It highlights the features, research progress, and clinical potential of these drugs, linking them to transporters like LRP1, RAGE, and AQP4, and the brain-peripheral linkage theory, offering insights for future AD drug development and clinical application.

### 4.1 Targeting transport pathways and lymphatic system regulation

The core role of such drugs is to repair the integrity of the brain-peripheral Aβ transport pathway and enhance lymphatic drainage function, thereby improving the transport efficiency of Aβ from the brain to the periphery, creating prerequisites for the clearance of Aβ by peripheral organs (liver, kidneys, *etc.*). At the same time, some drugs can synergistically improve the brain delivery efficiency of other anti-AD drugs or Aβ clearance effects, having important combined therapeutic value (Table 3).

**TABLE 3:** Brain clearance and transport modulators (glymphatic/lymphatic/BBB).

Drug name	Target/Core pathway	Research stage	Core advantages	Core limitations	Ref.
Vascular Endothelial growth Factor C (VEGF-C)	VEGF-C/VEGFR-3 signaling pathway, dural lymphatic vessels, neural stem cells	Preclinical research stage (only animal experiments carried out, not entered clinical trials)	Specifically targets the Aβ brain-peripheral lymphatic transport pathway, with triple effects of promoting dural Lymphangiogenesis, hippocampal neurogenesis, and enhancing Aβ transport1; combined with Aβ Monoclonal antibodies can significantly improve Aβ clearance efficiency	Lacks an efficient brain-targeted delivery system, and the local Bioavailability in the brain needs to be improved; has not entered clinical trials, and the clinical transformation value of lymphatic drainage Regulation needs further verification	Louveau et al. (2015), Calvo et al. (2011)
Perillyl Alcohol (natural monoterpene compound)	Tight junctions of blood-brain barrier (BBB) endothelial cells	Preclinical research stage (positioned as a combination drug synergist, no clinical trials for AD Treatment alone)	Natural origin, good biosafety; can reversibly regulate the permeability of tight junctions of BBB endothelial cells, significantly improve the brain delivery efficiency of therapeutic drugs, and provide ideas for combined treatment.	No independent anti-AD therapeutic activity, only used as a drug delivery adjuvant; the optimal dose ratio, administration timing, and long-term safety of clinical combined Medication still need systematic exploration	Wang et al. (2021a)
TGN-073	Aquaporin 4 (AQP4), glymphatic system	Preclinical research stage (only used for animal experiments and basic research, no clinical trials initiated)	It directly regulates AQP4, the core molecule of the glymphatic system, Promotes cerebrospinal fluid (CSF)-interstitial fluid (ISF) convection, and accelerates intracerebral Aβ drainage. It also indirectly preserves neurovascular unit integrity and mitigates Aβ-induced vasogenic edema.	The activating effect on AQP4 is mild, and the evidence for its Cognitive improvement effect in AD model animals is insufficient; long- term administration safety data is Lacking, and it has not entered the clinical development process	Alghanimy et al. (2023)
OAB-14	PPARγ–P2X7R–A QP4 pathway, microglia, IDE, NEP, endosome- autophagy-lysosome pathway	Clinical trial stage (clinical research in progress, data not yet fully disclosed)	It mediates multi-target synergistic regulation, exerting glymphatic drainage enhancement, accelerated Aβ clearance, and neuroprotection. With favorable Blood-brain barrier permeability, its mechanism—dominated by boosted Clearance rather than Aβ production inhibition—differs from conventional failed agents, conferring substantial clinical translational potential	The sample size and follow-up period of clinical trials are limited, and the long-term efficacy and safety need to be verified by large-scale multi-center studies; the applicable population (such as AD stage, genotype stratification) has not been clearly defined.	Alghanimy et al. (2023); Zhang X. et al. (2023); Yuan et al. (2019); Zheng et al. (2025)

#### 4.1.1 Vascular endothelial growth factor C (VEGF-C)

Core role: Focuses on the dual regulation of dural lymphangiogenesis (Louveau et al., 2015) and neurogenesis, specifically enhancing the function of the lymphatic pathway for Aβ brain-peripheral transport, providing a new target for early AD intervention.

Compound classification: Recombinant engineered protein (artificially bio-synthesized molecule).

FDA approval status for AD: No FDA approval for Alzheimer’s disease; only preclinical animal research, no clinical trial applications submitted.

Mechanism of action: 1) The VEGF-C/VEGFR-3 signaling pathway is a key regulatory pathway for neural stem cell activation and proliferation (Calvo et al., 2011). VEGFR3 gene deficiency can completely block neurogenesis. Supplementing exogenous VEGF-C in AD animal models can significantly promote hippocampal neurogenesis and improve cognitive decline (Da Mesquita et al., 2018); 2) Experiments on aged mice have confirmed that modified VEGF-C (mVEGF-C) can significantly enhance the drainage capacity of the dural lymphatic system. When combined with Aβ monoclonal antibodies, it can greatly improve the intracerebral Aβ clearance rate through the synergistic effect of “transport enhancement + specific clearance”, improving the “transport-clearance” synergistic network (Da et al., 2021); 3) Indirectly regulates the homeostasis of the intracerebral microenvironment, reduces neuroinflammation and oxidative stress, and provides favorable conditions for the functional repair of BBB transporters (such as LRP1).

Research update: The drug is in preclinical stages, having been tested only on animals. It has shown promising results in enhancing lymphatic drainage and neurogenesis in Alzheimer’s models, suggesting a new path for early intervention. Future studies should aim to optimize brain-targeted delivery to boost local bioavailability, paving the way for clinical trials.

#### 4.1.2 Perillyl alcohol

Core role: As a natural monoterpene compound, its core function is a BBB opening modulator, which can improve the brain delivery efficiency of anti-AD drugs by reversibly regulating BBB permeability, and indirectly enhancing the regulatory effect of the Aβ brain-peripheral transport pathway (Wang et al., 2021a).

Compound classification: Natural small-molecule compound (naturally extracted from citrus, mint, and other plants; chemical synthetic preparation is also available).

FDA approval status for AD: Not approved by FDA for Alzheimer’s disease; its purified formulation NEO100 has entered Phase II trials for recurrent glioblastoma, with no AD clinical development filings.

Mechanism of action: By specifically regulating the expression and distribution of tight junction-related proteins (such as occludin and claudin-5) in BBB endothelial cells, it reversibly increases BBB permeability, significantly improving the brain delivery efficiency of drugs such as VEGF-C and trastuzumab (Wang et al., 2021b), effectively solving the key technical bottleneck of insufficient brain penetration of such drugs, and providing important ideas for the synergistic application of transport pathway regulatory drugs and Aβ clearance drugs. This process is time-sensitive and reversible, without obvious central neurotoxicity. Research update: Animal studies have validated the safety and efficacy of its BBB opening effect. It is primarily used as a synergist with other anti-AD drugs to enhance effectiveness, with no standalone AD clinical trials. Current efforts aim to optimize combination regimens (dose ratio, timing) and confirm its synergistic effects in preclinical models, supporting future clinical applications.

### 4.2 Targeting AQP4 and the glymphatic system

Glymphatic System dysfunction is an early factor in Aβ buildup in the brain, as it clears metabolic waste via CSF-ISF convection. Certain drugs improve this system’s efficiency by targeting aquaporin 4 (AQP4) to enhance Aβ drainage, while others offer broader neuroprotective benefits for AD.

#### 4.2.1 TGN-073

Core role: As a specific AQP4 function promoter, it focuses on improving the drainage efficiency of the glymphatic system, accelerating intracerebral Aβ clearance, and maintaining the homeostasis of the intracerebral microenvironment.

Compound classification: Artificially synthesized small-molecule compound.

FDA approval status for AD: No FDA approval for Alzheimer’s disease; only basic research and animal experiments, no clinical development plans launched.

Mechanism of action: 1) Directly activates the water transport function of AQP4, promotes CSF-ISF convection efficiency, accelerates the drainage of intracerebral metabolic wastes such as Aβ to perivascular spaces, and reduces Aβ deposition in the brain (Alghanimy et al., 2023); 2) Theoretically, it can protect the integrity of the neurovascular unit by regulating the water homeostasis of astrocyte endfeet, reduce Aβ-induced vasogenic edema, indirectly maintain BBB barrier function, and provide a stable structural guarantee for Aβ brain-peripheral transport.

Research progress: Currently, the drug is only used for basic research and animal experiments, and clinical trials have not been initiated, nor has it entered the clinical development process. Its activating effect on AQP4 is relatively mild, and the evidence for its cognitive improvement effect in AD model animals is insufficient, and long-term administration safety data is lacking. Subsequent studies need to further enhance its strength and specificity to provide support for clinical transformation (Figure 6).

**FIGURE 6:**
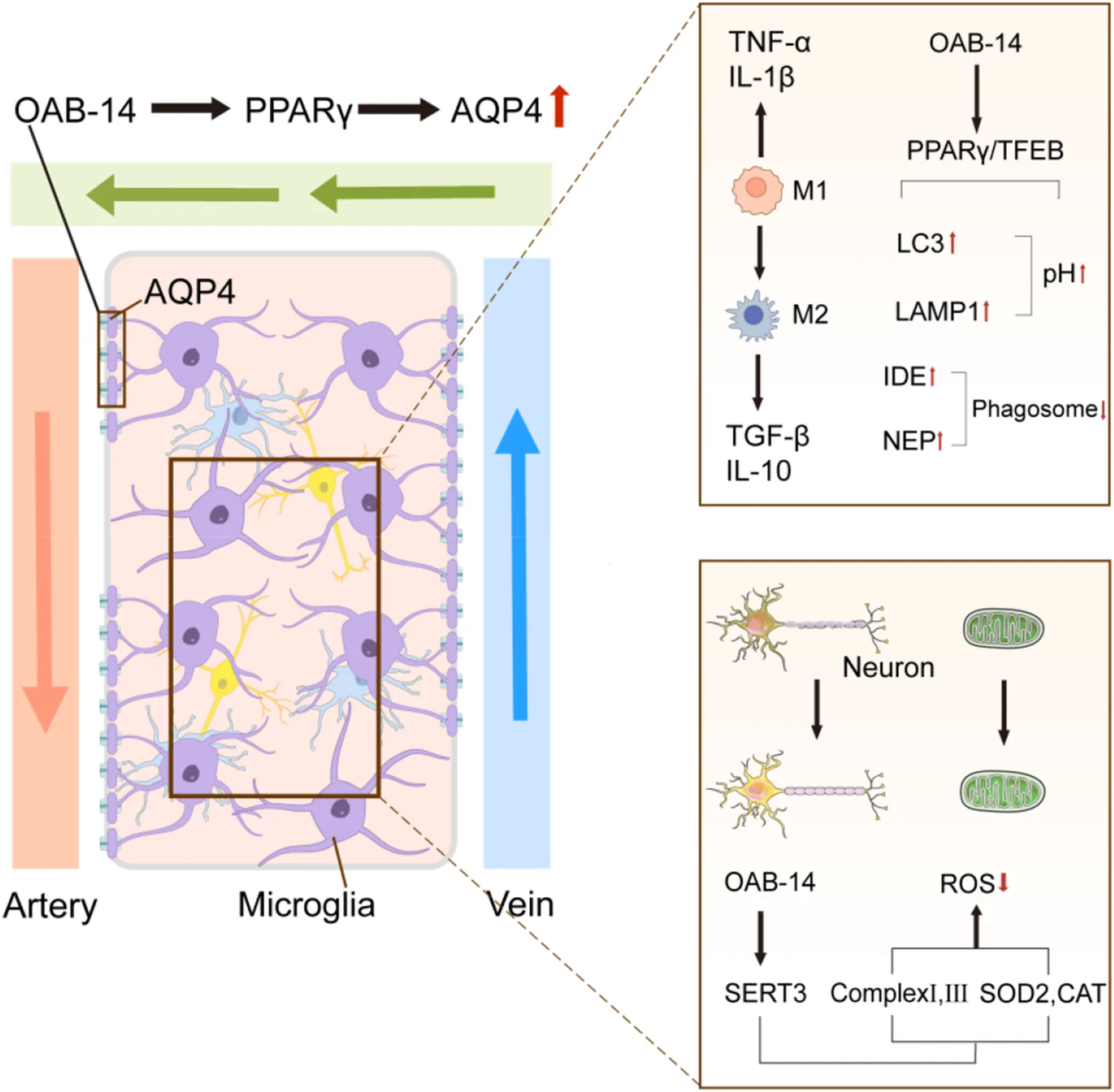
Schematic of the main therapeutic mechanisms of the small molecule OAB-14 in Alzheimer’s disease (AD).1. Activates the PPARγ/P2XTR pathway to upregulate AQP4 expression at astrocyte endfeet, promoting CSF-ISF circulation to clear Aβ oligomers and plaques.2. Regulates microglial gene expression via PPARγ to promote IDE and NEP synthesis for degrading Aβ into non-aggregating short peptides; upregulates LC3 and LAMP1 to enhance microglial autophagy and lysosome biogenesis.3. Activates SIRT3 to repair damaged mitochondria, and deacetylates/activates SOD2 to reduce ROS production, thereby resisting neuronal oxidative damage. OAB-14 exerts comprehensive and highly efficient therapeutic effects on AD via multiple pathways.

#### 4.2.2 OAB-14

Core role: A multi-target Glymphatic System regulatory drug with multiple functions of glymphatic drainage enhancement, Aβ clearance acceleration, and neuroprotection. It is one of the core candidate drugs for clinical transformation of glymphatic-targeted drugs.

Compound classification: Artificially synthesized small-molecule compound.

FDA approval status for AD: Not approved by the FDA for Alzheimer’s disease; stays in the preclinical animal research stage without a clinical trial application.

Mechanism of action: 1) Upregulates the expression and localization of AQP4 in astrocyte endfeet through the PPARγ-P2X7R-AQP4 signaling pathway (Zhang X. et al., 2023), significantly enhancing the drainage function of the glymphatic system; 2) Promotes the transformation of microglia to the protective phenotype (M2 type), enhances their phagocytic activity, and simultaneously upregulates the expression of key Aβ-degrading enzymes (insulin-degrading enzyme IDE, neutral endopeptidase NEP), achieving “dual-pathway enhancement” of Aβ clearance (glymphatic drainage + enzymatic degradation) (Yuan et al., 2019); 3) Has significant central anti-inflammatory, antioxidant, and anti-neuronal apoptosis effects, which can effectively improve the neuroinflammatory microenvironment induced by Aβ (Yuan et al., 2019); 4) Repairs mitochondrial function damage through the SIRT3-dependent mechanism, regulates the homeostasis of the endosome-autophagy-lysosome pathway, promotes receptor-mediated endocytosis, and restores the clearance capacity of the autophagy pathway for Aβ (Zheng et al., 2025; Guo et al., 2021); 5) Different from traditional anti-AD drugs, its mechanism of action is mainly “clearance enhancement” without inhibiting the activity of Aβ-producing enzymes (such as BACE1, γ-secretase) (Zhang X. et al., 2023), avoiding the neurotoxicity risk caused by excessive inhibition of Aβ production.

Research progress: The drug has good BBB penetration and is currently in the clinical trial stage (data not yet fully disclosed). Its unique multi-target mechanism of action and excellent pharmacokinetic characteristics provide an important reference for the clinical transformation of glymphatic system-targeted drugs. Subsequent studies need to focus on the efficacy and safety data of its large-scale clinical trials.

### 4.3 Targeting microglial function regulation

As the intrinsic immune cells of the central nervous system, abnormal phagocytic function of microglia is closely related to Aβ accumulation in the brain. Such drugs specifically regulate the activation state of microglia (rather than simple proliferation), enhance their phagocytic clearance capacity for Aβ, and avoid neuroinflammatory damage induced by excessive activation, achieving balanced regulation of “clearance-anti-inflammation” (Table 4).

**TABLE 4:** Microglia and neuroinflammation modulators.

Drug name	Target/Core pathway	Research stage	Core advantages	Core limitations	Ref.
VG-3927 (and its new generation Derivative C1)	Triggering receptor expressed on myeloid cells 2 (TREM2) signaling pathway, microglia	VG-3927: Phase I clinical research (SAD/MAD trials completed); Derivative C1 Preclinical research stage	It is the first small-molecule TREM2 agonist to enter clinical trials, with high oral bioavailability and excellent intracerebral targeting; it specifically activates the protective phenotype of microglia, enhances Aβ/tau Phagocytic clearance without inducing excessive neuroinflammation, and its derivative C1 has higher activity than the parent drug	The phase II clinical efficacy has not been verified, and it remains unclear Whether it can significantly improve the cognitive function of AD patients Additionally, potential risks—such as abnormal glial cell proliferation induced by long-term activation of TREM2 Signaling—require systematic evaluation	Mirescu et al. (2024)
Curcumin	Aβ, tau protein, and microglia (regulating M1/M2 phenotypic switch)	*In vitro*/animal experiment stage; research on optimization by nano-carrier Modification in progress (no Clinical trials initiated)	Natural product origin, significant multi-target advantages, can Simultaneously exert Aβ aggregation inhibition, tau hyperphosphorylation Blocking, anti-inflammation, anti-oxidation, and neuroprotection effects; After modification by nano-carriers (solid lipid nanocapsules, lipid-core nanocapsules), brain targeting and bioavailability are significantly Improved	Inherently extremely low water solubility (only 11 ng/mL in pH 5.0 buffer), poor chemical stability, and extremely low oral bioavailability; Unmodified curcumin cannot reach the effective therapeutic concentration in the brain, and clinical transformation Depends on the optimization of delivery systems; no clinical trials have been Initiated	Ege (2021); Jakubowski et al. (2020); Wu X. et al. (2022)
AL0020	Microglial proliferation signaling pathway	Phase II clinical research failed (did not reach the main clinical endpoint, subsequent Development terminated)	Can effectively induce microglial proliferation in animal experiments, reduce the formation of filamentous Aβ plaques and neurite dystrophy in the brain, and has certain Aβ clearance potential.	Phase 2 MRI reported dose-dependent amyloid-related imaging abnormalities (ARIA): After excluding APOE ε4 homozygotes, ARIA-E (vasogenic Edema) occurred in 29% of high-dose recipients and ARIA-H (cerebral Microhemorrhage/hemosiderosis) in 27%, with higher incidence in APOE4 carriers; cases were mostly Asymptomatic but some were Accompanied by headache, confusion or other neurological symptoms, requiring Dose suspension or symptomatic management	Mummery et al. (2026)

#### 4.3.1 VG-3927 (and its new generation derivative C1)

Core role: As the first small-molecule triggering receptor expressed on myeloid cells 2 (TREM2) agonist to enter clinical trials, it can precisely regulate microglial function, achieving synergistic effects of Aβ/tau clearance and neuroinflammation inhibition, marking the transformation of AD treatment to a small-molecule precision regulation paradigm (Mirescu, 2024).

Compound classification: Artificially synthesized small-molecule compound; C1, its derivative, is also chemically synthesized.

FDA approval status for AD: No FDA approval for Alzheimer’s disease; only Phase I clinical data available, Phase II trials ongoing and uncompleted.

Mechanism of action: 1) Has good BBB penetration and oral bioavailability (Rajagovindan et al., 2024). By specifically activating the TREM2 signaling pathway, it induces the transformation of microglia to the protective phenotype, significantly enhancing their phagocytic clearance capacity for aggregated Aβ and tau protein, and simultaneously inhibiting the release of pro-inflammatory factors (TNFα, IL-1β, *etc.*) without inducing excessive neuroinflammation (Mirescu, 2024; Mirescu et al., 2024); 2) Compared with traditional Aβ monoclonal antibodies, its small-molecule characteristics and oral administration have obvious clinical application advantages, which can improve patient medication compliance and provide convenience for long-term AD treatment.

Research Update: Phase I trials of VG-3927 demonstrated good safety, with only mild to moderate adverse events that resolved on their own. The cerebrospinal fluid’s soluble TREM2 (sTREM2) levels decreased dose-dependently, supporting a once-daily oral dose. The derivative C1 shows enhanced TREM2 activation and remains in preclinical research. Future studies should verify VG-3927’s Phase II efficacy and its impact on cognitive function in Alzheimer’s patients.

#### 4.3.2 AL002

Core role: A microglial proliferation inducer that achieves Aβ plaque clearance by inducing microglial proliferation, its target regulation strategy has inherent defects, ultimately leading to the failure of clinical research.

Compound classification: Artificially engineered monoclonal antibody.

FDA approval status for AD: No FDA approval for Alzheimer’s disease; Phase II clinical trials failed to meet primary endpoints, further clinical development suspended (Mummery et al., 2026).

Mechanism of action: By activating the TREM2-DAP12-P13K/Akt proliferation signaling pathway of microglia, it increases the number of microglia in the brain. Theoretically, it can enhance Aβ phagocytic clearance efficiency and reduce the formation of filamentous Aβ plaques and neurite dystrophy (Long et al., 2024). However, the drug only focuses on the proliferation of microglial number, ignoring the specific regulation of their activation phenotype, leading to excessive activation of microglia (Ma et al., 2025). While inheriting the way of AL002 activating microglia, VG-3927 adds specific regulation to it, avoiding excessive activation of microglia and aggravation of neuroinflammation, and reducing treatment side effects.

Research progress: The Phase II INVOKE-2 trial did not achieve its primary goal or significantly slow early Alzheimer’s progression due to overactivated microglia worsening neuroinflammation. This highlights the need for future microglia-targeted drug development to focus on “phenotype regulation” rather than increasing microglia numbers, ensuring a balance between phagocytic clearance and anti-inflammatory effects (Ma et al., 2025) (Figure 7).

**FIGURE 7:**
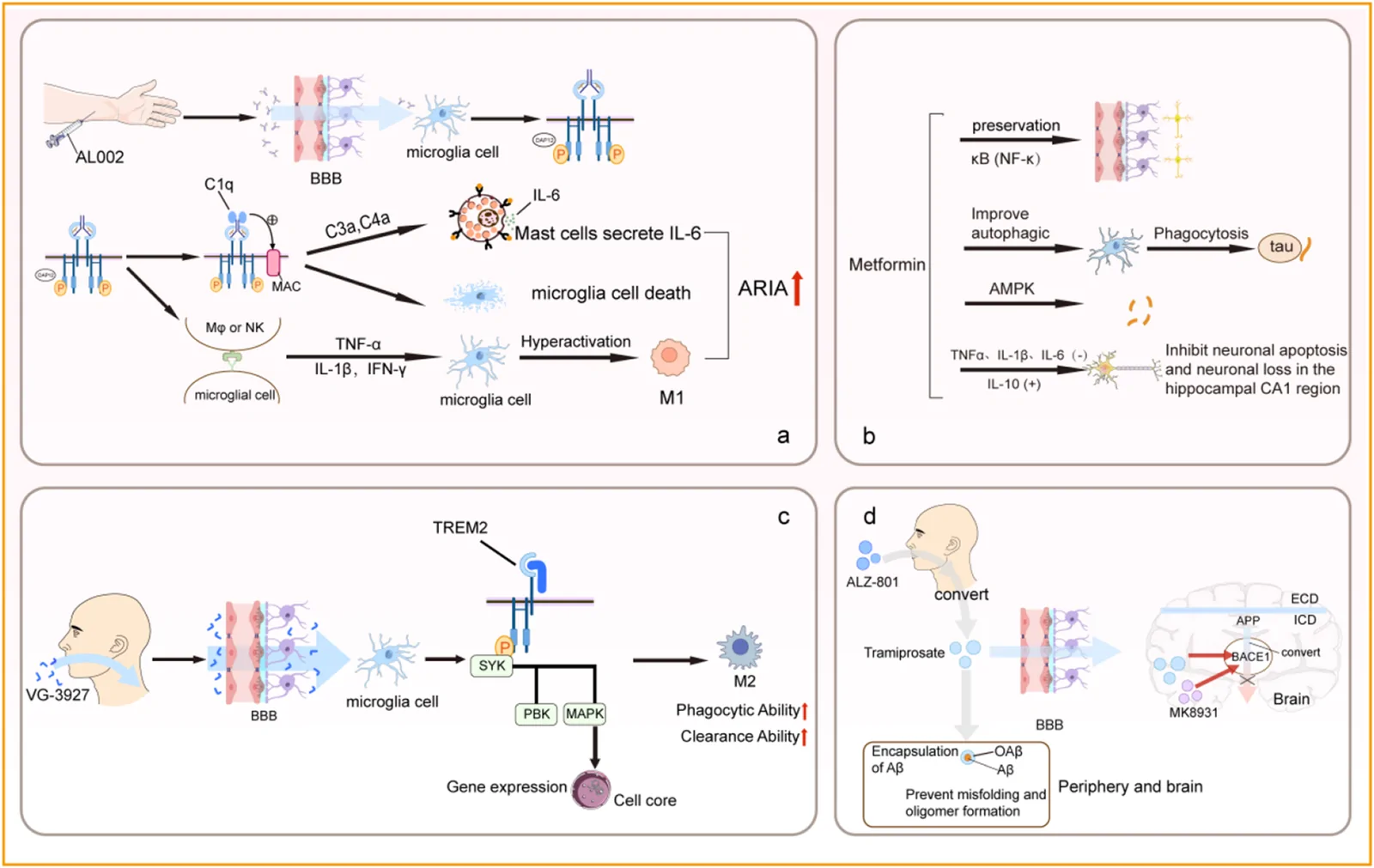
Schematic comparison of the limitations of clinical-stage anti-AD agents and the superior profiles of novel candidates. **(a)** Neuroinflammation and ARIA induced by AL002-mediated microglial M1 polarization. **(b)** Superior target affinity and Fc-independent safety profile of VG-3927 compared to AL002. **(c)** Multi-modal mechanisms of the multi-target agent metformin. **(d)** Modulatory effects of the ALZ-801“coating mechanism” on Aβ42 monomer stabilization and APP cleavage.

#### 4.3.3 Aducanumab

Core role: A humanized anti-Aβ monoclonal antibody targeting aggregated Aβ species, which eliminates cerebral Aβ plaques via microglia-mediated phagocytosis. Nevertheless, its non-selective immune activation and obvious safety risks limit its clinical application value.

Compound classification: Artificially bioengineered humanized monoclonal antibody, not natural compound.

FDA approval status for AD: Once granted accelerated approval for AD, later voluntarily withdrawn from the market due to inconsistent cognitive efficacy endpoints in Phase III trials; no active FDA-approved indication at present.

Mechanism of action: 1) Specifically binds to insoluble fibrillar Aβ deposited in brain parenchyma, triggers Fcγ receptor signaling on microglia, drives microglia to engulf and degrade Aβ plaques, and lowers cerebral Aβ load (Cadiz et al., 2024). Unlike small-molecule TREM2 agonists such as VG-3927, aducanumab cannot cross the BBB efficiently and requires high-dose intravenous infusion to reach effective intracerebral concentration; 2) Its non-specific activation of microglia tends to induce biased M1 pro-inflammatory polarization, easily triggering neuroinflammation, vasogenic edema and amyloid-related imaging abnormalities (ARIA), which is the core clinical limitation consistent with AL002s defective regulatory logic of overactivating microglia (Sevi et al., 2016). Research Update: Aducanumab once received accelerated FDA approval based on plaque-lowering endpoints, but two pivotal Phase III trials failed to reach the primary cognitive efficacy endpoint. High ARIA incidence in treated cohorts greatly restricted its clinical popularization, demonstrating that antibody-mediated microglial clearance without phenotype control carries prominent inflammatory toxic risks. Subsequent drug development has turned to optimizing antibody binding selectivity and microglial polarization balance.

#### 4.3.4 Lecanemab

Core role: A modified anti-Aβ protofibril monoclonal antibody with higher binding specificity for soluble toxic Aβ aggregates, which mediates microglial clearance of Aβ while partially mitigating the inflammatory safety hazards seen in aducanumab.

Compound classification: Artificially bioengineered humanized monoclonal antibody, not natural compound.

FDA approval status for AD: Fully FDA-approved for the treatment of early-stage Alzheimer’s disease.

Mechanism of action: 1) Preferentially recognizes soluble Aβ protofibrils rather than mature insoluble plaques, recruits peripheral and central microglia through Fc-dependent pathways to clear toxic Aβ oligomers and protofibrils, interrupting plaque deposition at an early stage of pathology (Kastanenka et al., 2016); 2) Compared with aducanumab, lecanemab reduces the incidence of severe ARIA to a certain extent by optimizing epitope binding, yet it still cannot avoid microglia overactivation-related inflammatory responses fundamentally. In contrast, small-molecule VG-3927 acts directly on the TREM2 pathway to induce protective anti-inflammatory microglial phenotypes without Fc-mediated immune stimulation, possessing superior safety advantages (Söderberg et al., 2023); 3) Beyond central clearance, lecanemab modulates peripheral circulating Aβ pools through the peripheral sink effect, assisting whole-body Aβ elimination. Research Update: Two pivotal Phase III trials confirmed its significant efficacy in slowing cognitive decline in early AD patients, securing full FDA approval. Infusion-related reactions and mild-to-moderate ARIA remain the main adverse events, requiring regular MRI monitoring during medication. Current research focuses on combining lecanemab with anti-inflammatory small molecules to balance microglial clearance efficiency and neuroinflammatory suppression.

#### 4.3.5 Donanemab

Core role: A plaque-targeted monoclonal antibody specific for N-terminal pyroglutamate-modified Aβ, which robustly clears mature cerebral amyloid plaques by activating microglial phagocytosis, with differentiated efficacy and safety profiles relative to lecanemab and aducanumab.

Compound classification: Artificially bioengineered humanized monoclonal antibody, not natural compound.

FDA approval status for AD: Received full FDA approval for early Alzheimer’s disease with confirmed amyloid pathology.

Mechanism of action: 1) Selectively binds pyroglutamate Aβ, a major component of mature neuritic plaques, activates microglial FcγR signaling to drive large-scale phagocytosis and degradation of compact Aβ plaques, achieving more thorough plaque removal than lecanemab (Al-Horani, 2024) a highertrong plaque clearance capacity is accompanied by higher ARIA risk than lecanemab, as extensive plaque breakdown triggers massive microglial activation and local inflammatory cascade. Similar to AL002 and aducanumab, donanemab only enhances microglial phagocytic activity without directional regulation of anti-inflammatory microglial phenotypes, resulting in unbalanced clearance-inflammation homeostasis (Manzano et al., 2020); 3) It exerts limited regulatory effects on soluble toxic Aβ protofibrils, showing complementary therapeutic characteristics to protofibril-targeted lecanemab. Research Update: Phase III clinical data demonstrated significant slowing of clinical and functional decline in early AD participants with moderate to high cerebral amyloid burden. ARIA rates are higher than lecanemab, and the clinical benefit-risk ratio requires individualized assessment according to amyloid load and cerebral vascular status. Ongoing studies explore dose adjustment and combined anti-inflammatory strategies to lower ARIA risk.

### 4.4 Aβ aggregation inhibitors and clearance enhancers

Such drugs directly act on the Aβ molecule itself, reducing the accumulation of toxic Aβ species (especially oligomers) in the brain by inhibiting their aggregation, promoting the depolymerization of formed fibers, or enhancing peripheral clearance; some drugs can synergize with the brain-peripheral transport pathway to improve the overall clearance efficiency, which is one of the traditional core directions in the research and development of AD therapeutic drugs (Table 5).

**TABLE 5:** Anti-Aβ agents (target Aβ production, aggregation and clearance).

Drug name	Target/Core pathway	Research stage	Core advantages	Core limitations	Ref.
Aducanumab	Aggregated Aβ (oligomers, insoluble Fibers/plaques), microglial Activation pathway	Withdrawn after FDA accelerated approval	Penetrates the blood-brain barrier, specifically binds Aβ plaques, and promotes microglia-mediated phagocytic clearance; dose-dependently reduces cerebral Aβ Burden and mitigates clinical decline	Controversial efficacy, high incidence of ARIA (edema/hemorrhage) (ARIA-E about 35%); failed to achieve stable clinical benefits and was eventually withdrawn from the market.	Cadiz et al. (2024); Sevi et al. (2016); Kastanenka et al. (2016); Söderberg et al. (2023)
Lecanemab	Soluble Aβ protofibrils, Microglial Fcγ Receptor (FcγRs) pathway	FDA fully approved, clinical application stage	Exhibits high affinity for neurotoxic Aβ protofibrils; dose-dependently preserves hippocampal volume, Slows cognitive decline by 26% and daily living impairment by 37% With a low ARIA-E incidence (∼12.6%) and favorable tolerability.	Requires intravenous infusion every 2 weeks, which affects patient Compliance; the risk of ARIA is Increased in APOEε4 carriers, and long-term efficacy and safety need further verification	Söderberg et al. (2023)
Tramiprosate (3-amino-1-propanesulfo nic acid)	Aβ monomers, glycosaminoglycan s, β-secretase 1 (BACE1)	Phase III clinical research (overall did not reach the main clinical Endpoint, positive in APOE4 gene carrier subgroup)	Combines central Aβ aggregation inhibition with a peripheral “sink” effect; competitively binds Aβ monomers to block aggregation and enhance peripheral clearance of cerebral Aβ. Its metabolite 3-SPA Also potently inhibits Aβ42 Aggregation, with good safety	The overall clinical research did not reach the preset main endpoint, and significant cognitive benefits were only observed in the APOE4 gene carrier subgroup, with a narrow applicable population; the Depolymerization effect on formed Aβ fibers is limited	Al-Horani (2024), Manzano et al. (2020)
ALZ-801 (valylproline)	Aβ monomers (prodrug, converted to tramiprosate after oral Administration)	Clinical development stage (phase I clinical completed, phase II clinical research in progress)	As a prodrug of tramiprosate, shows improved gastrointestinal absorption and tolerability; achieves ∼40% BBB penetration with low interindividual pharmacokinetic variability, and selectively targets toxic Aβ oligomers.	The efficacy depends on APOE4 genotype stratification, and the Clinical benefit of non-APOE4 gene carriers is not clear; large-scale phase II/III clinical studies are needed to verify its long-term efficacy and safety.	Al-Horani (2024), Kocis et al. (2017)

#### 4.4.1 Aggregation inhibitors

##### 4.4.1.1 Tramiprosate (3-amino-1-propanesulfonic acid)

Core role: A systemic pan-Aβ aggregation inhibitor that reduces intracerebral Aβ load through the synergistic effect of central aggregation inhibition and peripheral “sink effect”, especially having potential therapeutic value for apolipoprotein E4 (APOE4) gene carriers (Al-Horani, 2024).

Compound classification: Artificially synthesized small-molecule compound.

FDA approval status for AD: No FDA marketing approval for Alzheimer’s disease; only subgroup efficacy observed in terminated Phase III trials, no further FDA submission plans.

Mechanism of action: i. Specifically competitively binds to Aβ monomers, blocks their interaction with glycosaminoglycans, inhibits the formation of Aβ oligomers and fibers, without obvious tissue specificity; ii. Reduces the concentration of free Aβ in the peripheral blood, promotes the reverse diffusion of intracerebral Aβ to the peripheral circulation through the “peripheral sink effect”, and metabolizes and clears it through organs such as the liver and kidneys; iii. When reaching an effective concentration locally in the brain, it can slightly inhibit the activity of β-secretase 1 (BACE1), reducing the cleavage and processing of amyloid precursor protein (APP) into Aβ (Al-Horani, 2024; Manzano et al., 2020).

Research progress: The overall Phase III clinical trial of the drug failed to meet the preset primary endpoint, but significant cognitive benefits were observed in the APOE4 gene carrier subgroup, which can effectively prevent hippocampal atrophy and enhance cognitive function. Its metabolite 3-thiopropionic acid (3-SPA) also has high-efficiency Aβ42 aggregation inhibitory activity, and the severity of cognitive impairment in AD patients is negatively correlated with the concentration of 3-SPA in the cerebrospinal fluid. Currently, the clinical application of the drug is positioned as a precision treatment candidate for APOE4 gene-positive AD patients.

#### 4.4.2 ALZ-801 (valylproline)

Core role: As a prodrug of Tramiprosate, it optimizes gastrointestinal absorption efficiency and tolerability through structural modification, specifically targets toxic Aβ oligomers, and reduces the risk of adverse reactions in AD treatment (Al-Horani, 2024).

Compound classification: Artificially synthesized small-molecule prodrug.

FDA approval status for AD: No FDA approval for AD; currently undergoing Phase II clinical trials, no FDA review application filed (Hey et al., 2024; Hey et al., 2025).

Mechanism of action: After oral administration, it is specifically converted into Tramiprosate in the body, stabilizes the half-cyclic conformation of Aβ42 monomers through a “coating mechanism”, preventing their misfolding and oligomer formation; this process is highly selective and does not affect insoluble amyloid plaques or fibers already formed in the brain, avoiding the risk of toxicity diffusion caused by plaque dissociation (Al-Horani, 2024; Kocis et al., 2017).

Research progress: Currently, the drug is in the clinical development stage. The results of Phase I clinical trials confirmed that the plasma exposure of 265 mg twice daily is equivalent to that of Tramiprosate 150 mg twice daily, the BBB penetration rate is about 40%, the inter-individual pharmacokinetic variability is low, and the gastrointestinal tolerability is significantly better than that of the parent drug Tramiprosate. Subsequent Phase II clinical trials are being promoted, focusing on verifying its cognitive improvement efficacy in APOE4 gene-positive AD patients.

#### 4.4.3 Scyllo-Inositol

Core role: Specifically binds to Aβ42 and inhibits its fibrillation, having both Aβ aggregation prevention and pathological reversal effects, and is a potential dual candidate drug for AD prevention and treatment.

Compound classification: Natural endogenous small molecule (naturally present in human tissues; chemical synthetic production is also available).

FDA approval status for AD: No FDA approval for Alzheimer’s disease; remains in preclinical animal research with no clinical trial initiation.

Mechanism of action: It can efficiently penetrate the BBB, regulate its secondary structure conformation by specifically binding to the hydrophobic region of Aβ42, stabilize non-toxic Aβ42 monomers, completely block its fiber formation; at the same time, it can depolymerize formed Aβ fibers and reverse intracerebral Aβ pathological deposition; in addition, it can slightly upregulate the expression of IDE in the brain and accelerate Aβ enzymatic degradation (Sun et al., 2008).

Research progress: Animal experiments have confirmed that oral Scyllo-Inositol can significantly inhibit Aβ aggregation, reduce spatial memory impairment in AD model animals, and reduce intracerebral Aβ load, and can play a role whether administered before or after the appearance of AD-like phenotypes, showing good prevention and treatment potential. Currently, the drug is still in the preclinical research stage, the molecular mechanism of the Aβ42 binding site has not been fully clarified, and the target binding specificity needs further verification, and clinical trials have not been initiated.

#### 4.4.4 Clearance enhancer: MK8931 (verubecestat)

Core role: A potent, highly selective BACE1 inhibitor that significantly reduces intracerebral Aβ deposition by blocking the rate-limiting step of Aβ production, and is one of the representative candidate drugs of Aβ production inhibition drugs.

Compound classification: Artificially synthesized small-molecule compound.

FDA approval status for AD: No FDA approval for AD; all clinical development terminated after negative Phase III trial results (Egan et al., 2018; Egan et al., 2019).

Mechanism of action: Binds with high affinity and specificity to the hydrophobic subsite of the BACE1 active site, directly inhibits its catalytic activity, blocks the β-cleavage process of APP (the rate-limiting step of Aβ production), thereby reducing the production and deposition of β-CTF (CTFβ) and subsequent Aβ40/Aβ42, reducing intracerebral Aβ load (Forman et al., 2019).

Research progress: Preclinical studies have confirmed that oral MK8931 can potently and continuously reduce Aβ levels in cerebrospinal fluid and brain. At doses of 3–10 mg/kg, the decrease in cerebrospinal fluid Aβ40 reaches 72%–81%, and the effect lasts for more than 24 h, supporting once-daily oral administration. Phase III clinical trials of the drug have been completed, but due to potential neurotoxicity (such as cognitive deterioration and psychiatric symptoms) induced by long-term administration, the clinical benefit-risk ratio did not meet expectations, and subsequent development has been terminated. This study suggests that the therapeutic strategy focusing solely on Aβ production inhibition has limitations, and it is necessary to take into account Aβ transport and clearance links to achieve multi-dimensional regulation.

### 4.5 Multi-target neuroprotective drugs

The pathophysiology of AD involves complex, interconnected damage, making single-target drugs insufficient for reversing its progression. Multi-target drugs that address inflammation, oxidative stress, autophagy, Aβ clearance, transport functions, and neuronal survival better align with AD’s systemic pathology and hold greater clinical promise (Bassendine et al., 2020; Sagare et al., 2012; Li et al., 2018; Liu et al., 2024; Alsubaie et al., 2022).

#### 4.5.1 Metformin

Core role: As a clinically commonly used drug for the treatment of type 2 diabetes, it has significant value in repurposing, can exert anti-AD effects through multi-target synergistic regulation, and has multiple functions of Aβ clearance enhancement, anti-inflammation, anti-oxidation, and neuroprotection, and its safety has been fully verified.

Compound classification: Artificially synthesized small-molecule compound.

FDA approval status for AD: No specific FDA approval for Alzheimer’s disease; approved for type 2 diabetes; multiple off-label clinical trials for AD/MCI are ongoing (Luchsinger et al., 2016).

Mechanism of action: 1) Activates the adenosine monophosphate-activated protein kinase (AMPK) signaling pathway, upregulates the expression of Aβ-degrading enzyme IDE in the brain, accelerates Aβ clearance, and slightly reduces BACE1 expression to reduce Aβ production, achieving balanced regulation of Aβ metabolism (Chen et al., 2021); 2) Improves microglial autophagic function defects, promotes their phagocytic clearance of pathological Aβ and tau protein, and reduces Aβ deposition and neurofibrillary tangles (NP tau) formation in APP/PS1 mice (Chen et al., 2021); 3) Significantly reduces oxidative stress damage (decreases malondialdehyde MDA and reactive oxygen species ROS levels, increases superoxide dismutase SOD activity) and neuroinflammatory response (downregulates pro-inflammatory factors such as TNFα, IL-1β, IL-6, and upregulates anti-inflammatory factor IL-10 expression) in the hippocampus, inhibits neuronal apoptosis and neuronal loss in the hippocampal CA1 region (Lu et al., 2020; Khaleghi-Mehr et al., 2023); 4) Indirectly improves intracerebral insulin sensitivity, inhibits the activation of nuclear factor κB (NF-κB) signaling pathway, and protects the integrity of BBB and neurovascular units.

Research progress: Animal experiments have confirmed that 200 mg/kg/d is the optimal effective dose of metformin for anti-AD, which can significantly improve the learning and memory functions of AD model animals such as APP/PS1. Currently, multiple clinical studies are exploring its intervention effect in AD and mild cognitive impairment (MCI) stages, providing a new candidate regimen for early AD intervention. Its core advantages are clear safety, low cost, and multi-target synergy, but its brain targeting is insufficient, the effective therapeutic concentration in the brain is low, and the optimal dose and start time of clinical intervention still need to be further clarified.

#### 4.5.2 Curcumin

Core role: A natural polyphenolic compound with significant multi-target anti-AD activity, which can simultaneously exert Aβ aggregation inhibition, tau phosphorylation blocking, anti-inflammation, anti-oxidation, and neuroprotection effects, but its clinical application is limited by the inherent defect of extremely low oral bioavailability.

Compound classification: Natural small-molecule polyphenol extracted from turmeric; chemical synthetic products are also available.

FDA approval status for AD: No FDA approval for Alzheimer’s disease; only used as health supplement, no completed pivotal clinical trials for AD treatment.

Mechanism of action: 1) Specifically binds to Aβ40/Aβ42, destroys their β-sheet structure, inhibits the formation of Aβ oligomers and fibers, and can depolymerize formed Aβ fibers to reduce Aβ toxicity; slightly upregulates the expression of IDE and NEP in the brain to enhance Aβ enzymatic degradation (Ege, 2021); 2) Regulates the M1/M2 phenotypic switch of microglia, inhibits their excessive activation, reduces the release of pro-inflammatory factors (IL-1β, IL-6, TNFα), upregulates the expression of brain-derived neurotrophic factor (BDNF), and reduces neuroinflammatory damage (Jakubowski et al., 2020); 3) Clears ROS in the body, increases the activity of antioxidant enzymes such as SOD and catalase, reduces lipid peroxidation (decreases MDA levels), and protects the integrity of mitochondrial function (Wu X. et al., 2022); 4) Inhibits excessive phosphorylation of tau protein through the PTEN/Akt/GSK-3β signaling pathway, and simultaneously binds with high affinity to target proteins such as colony-stimulating factor 1 receptor (CSF1R), advanced glycation end product receptor (AGER), and NFKB1, regulates multiple downstream pathological pathways, and achieves systematic neuroprotection (Wu X. et al., 2022).

Research progress: *In vitro* experiments and animal experiments have confirmed that curcumin has significant anti-AD activity, but due to its inherent extremely low water solubility (only 11 ng/mL in pH 5.0 buffer), poor chemical stability, and strong first-pass effect after oral administration, its bioavailability is extremely low, limiting its clinical application. Current research focuses on optimizing its pharmacokinetic characteristics through modification with nano-carriers (such as solid lipid nanocapsules, lipid-core nanocapsules, nanomicelles, etc.). Curcumin modified by nano-carriers has significantly improved brain targeting and bioavailability. In AD model animals, 50 mg.

#### 4.5.3 Atorvastatin

Core role: A clinically mature synthetic small-molecule lipid-lowering statin with prominent systemic multi-target neuroprotective activity for AD repurposing. It exerts dual regulatory effects on the peripheral liver-brain Aβ clearance axis and central nervous system pathological lesions via pleiotropic pathways, combining Aβ elimination, anti-inflammation, anti-oxidation, tau phosphorylation inhibition and BBB protection, while its clinical benefits are highly dependent on patient lipid status and APOE genotype (Lu et al., 2020).

Compound classification: Artificially synthesized small-molecule compound.

FDA approval status for AD: No dedicated FDA approval for Alzheimer’s disease; FDA-approved for hyperlipidemia, retrospective cohort studies support its potential AD prevention value, large prospective trials yield inconsistent results.

Mechanism of action:Peripheral clearance regulation centered on hepatic LRP1 upregulation. It activates hepatic SREBP-2 signaling to upregulate liver LRP1 expression, accelerating uptake and degradation of circulating free Aβ and reinforcing the peripheral sink mechanism to restore balanced brain-blood Aβ gradient. It also weakly binds the hydrophobic region of Aβ1-42 to suppress plasma Aβ oligomerization and cut down toxic circulating aggregates refluxing into the brain parenchyma (Donahue et al., 2006; Khaleghi-Mehr et al., 2023).

### 4.6 Ultrasound-mediated BBB open to improve Aβ transport and clearance

Focused Ultrasound (FUS)-mediated blood-brain barrier opening technology provides an innovative physical intervention strategy for Alzheimer’s disease. The core mechanism of this method is that FUS can open the blood-brain barrier (BBB) in a controllable, reversible and safe manner, creating a key window for intervening in neurodegenerative diseases (Rezai et al., 2020). Under magnetic resonance guidance, combining low-intensity focused ultrasound with intravenously injected microbubbles, the microbubbles oscillate in the target area through acoustic cavitation effect, which can transiently and accurately increase the permeability of the blood-brain barrier. Our research found that this opening can be completely restored and closed within 24 h, showing good biosafety (Lipsman et al., 2018). It is worth noting that in the pathological process of Alzheimer’s disease, the BBB is not completely damaged, but presents a complex state of functional abnormalities. Therefore, this active and controllable opening strategy provides a necessary and flexible means to break through the bottleneck of drug delivery3.

Firstly, as a powerful drug delivery platform, this technology can effectively break through the barrier of drug delivery into the brain and significantly improve the bioavailability of therapeutic drugs in the brain. Clinical studies have shown that the combined application of FUS-mediated BBB opening and the anti-Aβ antibody aducanumab can achieve better Aβ clearance effect in the target brain region than the single use of aducanumab (Aljehani et al., 2024). Experimental research by Gerhard Leinenga et al. quantified this advantage, confirming that after opening the BBB with Scanning Ultrasound (SUS, an application form of FUS), the concentration of antibodies in the brain can be increased by up to 19 times (Leinenga et al., 2021). More cutting-edge delivery strategies also include loading nano-drugs (such as quercetin-modified sulfur nanoparticles) into the microbubble shell, realizing targeted and site-specific drug release triggered by ultrasound, thus showing higher delivery efficiency and therapeutic potential in the preclinical stage6.

In addition to serving as a drug delivery carrier, focused ultrasound itself may also have independent therapeutic effects that do not rely on exogenous drugs. In multiple animal experiments, only through repeated and controllable ultrasound-mediated BBB opening, it can activate microglia, promote the clearance of Aβ plaques and tau protein aggregates, accompanied by the improvement of memory function (Leinenga et al., 2021). This process might activate the brain’s lymphatic drainage system, speeding up the removal of harmful proteins. Similar effects are seen with certain phototherapy treatments. Early clinical evidence indicates that focused ultrasound can improve cognitive test results, suggesting its potential to regulate neural activity (Scott and Klaus, 2024).

Current studies have consistently confirmed that FUS-mediated BBB opening combined with microbubbles has good reversibility and safety (Scott and Klaus, 2024). This technology provides a unique pathway for targeted delivery of drugs, genes and even stem cells to deep brain structures such as the hippocampus (Rezai et al., 2020). However, the extensive clinical transformation of this technology still faces important challenges: although the BBB opening itself has been proven feasible, this does not directly mean that therapeutic drugs (such as aducanumab) can reach the expected intracerebral penetration concentration and bring definite clinical benefits (Karthika et al., 2024). In the future, large-scale, standardized and long-term follow-up randomized controlled trials are needed to systematically evaluate the substantial impact of focused ultrasound on the pathological process and cognitive outcomes of Alzheimer’s disease, thus laying a solid foundation for the optimization of its therapeutic parameters and clinical application (Scott and Klaus, 2024; Karthika et al., 2024).

## 5 Exosomes-mediated Aβ transport and clearance

As extracellular vesicles with a diameter of about 30–200 nm, exosomes can mediate intercellular communication by carrying biologically active substances such as proteins and miRNAs, and play an important role in the pathological process of AD (Liu et al., 2022). Studies have found that exosomes can easily cross the blood-brain barrier, and the proteins on their surface can be engineered to achieve targeted drug delivery for pathology-related molecules such as amyloid precursor protein (APP) (Guo et al., 2020). Mesenchymal stem cell-derived exosomes (MSC-Exo) can mitigate Alzheimer’s disease pathology through several mechanisms: they encapsulate enzymes like neprilysin and insulin-degrading enzyme to clear Aβ plaques, regulate microglial polarization and astrocyte activation to reduce neuroinflammation, and enhance neuroprotection by increasing brain-derived neurotrophic factor (BDNF), promoting neurogenesis, improving synaptic plasticity, and reducing pro-apoptotic proteins (Abdi et al., 2025). Exosomes can also serve as delivery carriers for miRNAs, such as delivering miR-223 and miR-146a, to further regulate neuroinflammation and cell apoptosis (Guo et al., 2020).

It is worth noting that exosomes have a dual role in AD: in addition to participating in the clearance of pathological proteins, they may also mediate the spread and transmission of pathogenic factors such as Aβ and Tau (Kalluri and LeBleu, 2020), so it is necessary to finely regulate their functions. In terms of application, exosomes modified by engineering (such as Fe65 modification) can achieve targeted delivery of therapeutic drugs such as autophagy inducers, significantly improving the cognitive function and pathological characteristics of Alzheimer’s disease model mice (Iyaswamy et al., 2023). In addition, exosomes themselves can also serve as a source of biomarkers for AD diagnosis, and their proteomic characteristics are helpful for early disease screening163. Although in preclinical studies, the administration of exosomes through intraventricular injection, intravenous injection or nasal administration can improve the behavioral performance and pathological indicators of animal models (Abdi et al., 2025), and MSC-related therapies have shown good safety in clinical trials (Li and Bai, 2025), exosome therapy still faces many challenges, including insufficient standardization of isolation, unclear optimal administration route and dose, complex composition of contents, and potential side effects (Guo et al., 2020). With its multi-target therapeutic potential, engineering flexibility and potential as a diagnostic tool, exosomes provide a new research direction for the diagnosis and treatment of Alzheimer’s disease (Li and Bai, 2025).

## 6 Expert opinion

Until now, most existing studies focus in isolation on the central Aβ transport pathways or the clearance function of a single peripheral organ, lacking systematic integration of the full-chain synergistic regulatory mechanisms of Aβ from CNS transport to peripheral clearance. We summarize relevant drugs (central-penetrating, transport modulators, clearance regulators) and clarifies the core cause of poor drug efficacy: dysfunction of the Aβ-regulating “brain-peripheral crosstalk network” and insufficient targeting of transport-clearance synergy. By clarifying the functional characteristics, molecular mechanisms, research progress, and clinical transformation potential of each type of drug, this review establishes a close connection with the regulatory mechanisms of key transporters (such as LRP1, RAGE, AQP4) and the brain-peripheral linkage theory, thereby filling the gap in the integration of Aβ transport-clearance cascade mechanisms in current research, providing theoretical support for the research and development and clinical transformation of novel AD therapeutic drugs, and offering new ideas for solving the bottleneck of poor efficacy of current AD drugs.

Our integrated analysis demonstrates distinct organ-specific disparities in peripheral Aβ clearance: the liver serves as the predominant peripheral clearance organ, responsible for approximately 60%–65% of systemic Aβ elimination, whereas renal clearance accounts for only 5%–12% of total Aβ removal. Consistently, liver-targeted Aβ clearance strategies have dominated current peripheral anti-AD drug development, with abundant preclinical evidence and relatively mature translational prospects (Tsoy et al., 2024).

Nevertheless, despite its modest contribution to basal Aβ clearance, the renal clearance pathway cannot be overlooked or dismissed as therapeutically irrelevant. Although hepatic clearance undoubtedly represents the primary and most clinically actionable target for peripheral Aβ modulation, the renal pathway retains unique and promising therapeutic potential as a complementary regulatory axis. From the perspective of pathological compensation and multi-target synergistic intervention, renal Aβ clearance may assist in optimizing whole-body Aβ metabolic homeostasis and ameliorating Aβ deposition, particularly under pathological conditions where hepatic clearance function is compromised or saturated.
