# Correction: New insight into Alzheimer’s disease: small molecule-Aβ in the brain transport and peripheral organ clearance

**DOI:** 10.3389/fragi.2026.2029384

**Published:** 2026-10-29

**Authors:** Tianci Ma, Yongyi Li, Yuting Ding, Shijie Liu, Chuanhao Gao, Xiang Li, Zhiqian Tong, Yan Lv

**Affiliations:** 1 Clinical Medical College, Hainan Medical University, Haikou, Hainan, China; 2 Proactive Health and Aging Medicine of Clinical Research and Translational Center, Beijing Geriatric Hospital, Beijing, China; 3 Neurology Department, Hainan General Hospital, Affiliated Hainan Hospital of Hainan Medical University, Haikou, Hainan, China

**Keywords:** Alzheimer’s disease, brain-peripheral transport and 1 clearance, melecular mechanism, therapeutic drugs, **β**-amyloid (A**β**)

Affiliation Hainan General Hospital was omitted from the paper. This affiliation has now been added for author Yan Lv.

Affiliation Hainan Provincial People’s Hospital was erroneously included in the paper. This affiliation has now been removed for author Yan Lv.

The original version of this article has been updated.

